# Characterizing Tyrosine Phosphorylation Signaling in Lung Cancer Using SH2 Profiling

**DOI:** 10.1371/journal.pone.0013470

**Published:** 2010-10-19

**Authors:** Kazuya Machida, Steven Eschrich, Jiannong Li, Yun Bai, John Koomen, Bruce J. Mayer, Eric B. Haura

**Affiliations:** 1 Departments of Bioinformatics, H. Lee Moffitt Cancer Center and Research Institute, Tampa, Florida, United States of America; 2 Department of Thoracic Oncology, H. Lee Moffitt Cancer Center and Research Institute, Tampa, Florida, United States of America; 3 Deparment of Molecular Oncology, H. Lee Moffitt Cancer Center and Research Institute, Tampa, Florida, United States of America; 4 Raymond and Beverly Sackler Laboratory of Genetics and Molecular Medicine, Department of Genetics and Developmental Biology, University of Connecticut Health Center, Farmington, Connecticut, United States of America; National Cancer Institute, United States of America

## Abstract

**Background:**

Tyrosine kinases drive the proliferation and survival of many human cancers. Thus profiling the global state of tyrosine phosphorylation of a tumor is likely to provide a wealth of information that can be used to classify tumors for prognosis and prediction. However, the comprehensive analysis of tyrosine phosphorylation of large numbers of human cancer specimens is technically challenging using current methods.

**Methodology/Principal Findings:**

We used a phosphoproteomic method termed SH2 profiling to characterize the global state of phosphotyrosine (pTyr) signaling in human lung cancer cell lines. This method quantifies the phosphorylated binding sites for SH2 domains, which are used by cells to respond to changes in pTyr during signaling. Cells could be grouped based on SH2 binding patterns, with some clusters correlated with EGF receptor (EGFR) or K-RAS mutation status. Binding of specific SH2 domains, most prominently RAS pathway activators Grb2 and ShcA, correlated with EGFR mutation and sensitivity to the EGFR inhibitor erlotinib. SH2 binding patterns also reflected MET activation and could identify cells driven by multiple kinases. The pTyr responses of cells treated with kinase inhibitors provided evidence of distinct mechanisms of inhibition.

**Conclusions/Significance:**

This study illustrates the potential of modular protein domains and their proteomic binding profiles as powerful molecular diagnostic tools for tumor classification and biomarker identification.

## Introduction

Receptor and non-receptor tyrosine kinases regulate many activities important for cancer, including cell proliferation, survival, invasion/metastasis, and angiogenesis [1]. These signaling proteins therefore represent an important class of drug targets for the treatment of cancer, and numerous tyrosine kinase inhibitors (TKIs) are under development or are now being used in the clinic. Lung cancer accounts for over 160,000 deaths per year in the U.S. [2], so there is a powerful rationale to identify key drivers of lung cancer that can be therapeutically exploited. The activity of the epidermal growth factor receptor (EGFR) is frequently elevated in lung cancer, and inhibition of EGFR through the TKI erlotinib can extend survival in patients with advanced lung cancer refractory to chemotherapy [3]. In addition to EGFR, a number of other tyrosine kinases have been proposed as therapeutic targets in lung cancer, including MET, insulin-like growth factor receptors (IGFR), SRC kinases, fibroblast growth factor receptors (FGFR), platelet-derived growth factor receptors (PDGFR), anaplastic lymphoma kinase (ALK), and EPH receptors [4], [5], [6], [7], [8], [9], [10], [11], [12].

A key question in TKI therapy for lung cancer is which patients will benefit from these drugs, since the cost is substantial and many receive no benefit from treatment. An important breakthrough was the discovery of activating somatic mutations in EGFR that enhance receptor signaling and predict sensitivity to TKIs targeting the EGFR, such as erlotinib and gefitinib [13], [14], [15]. In lung cancer patients harboring these mutations, response rates to EGFR TKIs can be high and survival is better than that seen with cytotoxic agents [16]. Nonetheless some patients without EGFR mutation can benefit from EGFR inhibitors, and markers such as EGFR gene amplification, autocrine TGFα production, or gene expression profiles have been proposed to identify these patients [17], [18], [19]. In addition, resistance mechanisms such as MET amplification or secondary mutations in EGFR can rapidly lead to drug resistance [20], [21], [22]. Finally, some tumor cells are likely to be driven by multiple tyrosine kinases, and methods to identify and classify these are needed [23].

Proteomic strategies (which examine global patterns of protein expression or phosphorylation) are also being used to classify tumors [24]. Mass spectrometry (MS) coupled with anti-phosphotyrosine antibodies identified different patterns of tyrosine kinase signaling in lung cancer cells and tumors, and this approach was able to identify cells driven by oncogenic EGFR, PDGFR, and ALK [4]. Other studies using the same approach found patterns of tyrosine phosphorylation associated with mutant EGFR signaling [25], [26]. Overall, this work provides proof of principle that global tyrosine phosphorylation patterns can provide useful information for tumor classification. However, current MS methods require relatively large amounts of sample and quantification of phosphorylated sites is challenging, thus new phosphoproteomic strategies are needed.

We have developed an alternative phosphoproteomic method, termed SH2 profiling, that complements MS-based approaches [27], [28]. SH2 profiling is highly sensitive and throughput is relatively high, thus it is ideal for profiling phosphotyrosine (pTyr) signaling in cancer cells. The conceptual basis of SH2 profiling is to use the cell's own pTyr signal response apparatus to interrogate the state of pTyr signaling. Upon receptor tyrosine kinase (RTK) activation, the resulting increase in protein tyrosine phosphorylation generates binding sites for modular pTyr-specific binding domains; it is the relocalization of intracellular effectors containing pTyr binding domains to these phosphorylated sites that is the key step in signal transmission [29]. By far the most abundant pTyr binding module in humans is the Src Homology 2 or SH2 domain [30]. There are 120 SH2 domains encoded by the human genome, and each SH2 domain binds a unique spectrum of tyrosine phosphorylated sites [28], [31]. Because SH2 domains are what the cell uses to respond to or “read” changes in tyrosine phosphorylation during signaling, the extent of binding of different SH2 domains can provide a wealth of information about the mechanisms and status of pTyr signaling.

To test if this approach is useful in characterizing and classifying complex tumor types such as lung cancer, where multiple tyrosine kinases drive downstream signaling and maintain tumor growth, we applied SH2 profiling to lung cancer cell lines. We find that pTyr patterns could be related to known features of the cells including EGFR mutation status and sensitivity to EGFR TKI. Our results suggest that SH2 profiling provides novel insights into pTyr signaling that are likely to be useful for prediction and prognosis of lung cancer.

## Results

### SH2 profiling identifies subsets of lung cancer cell lines

**Figure 1:**
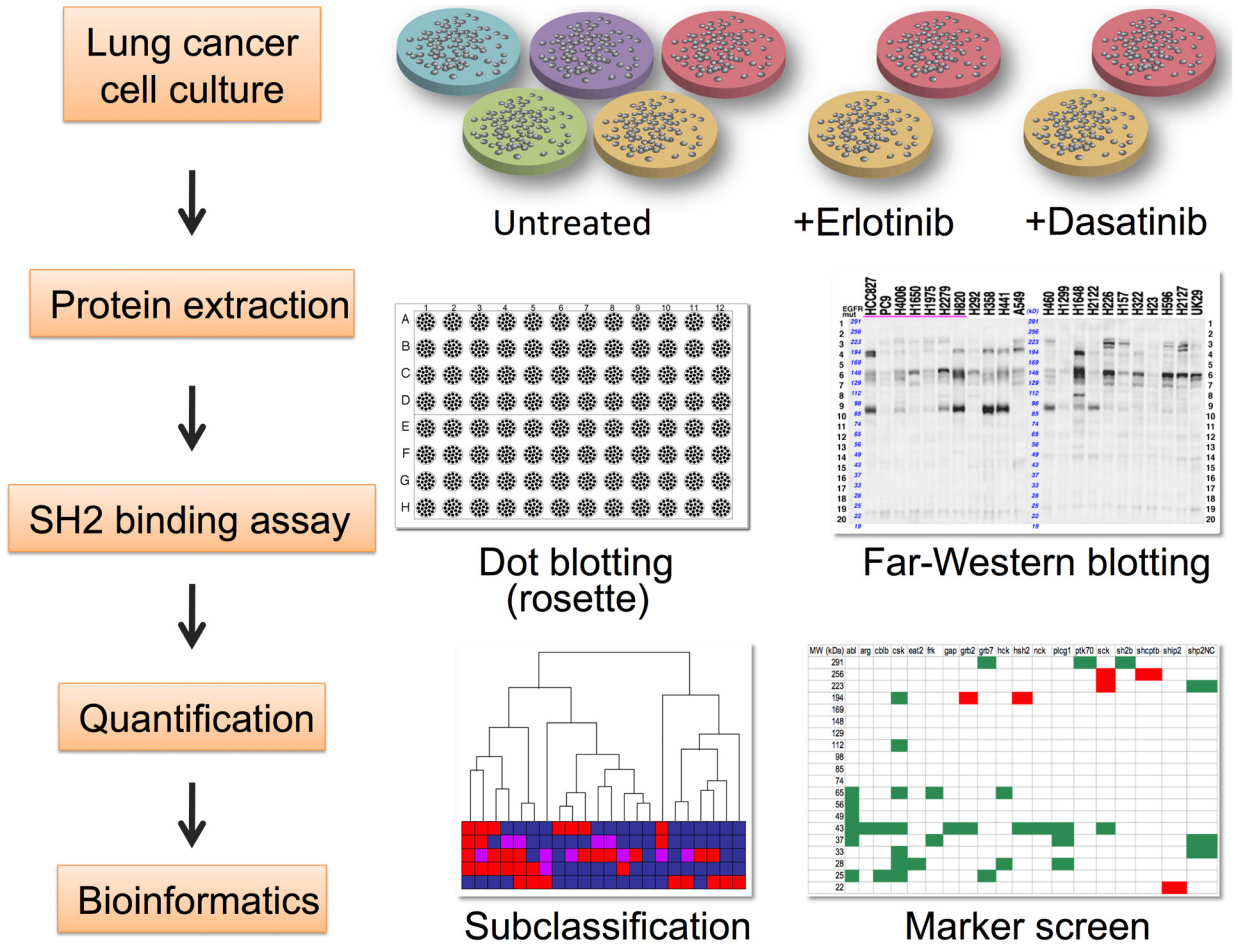
Overview of approach. Human lung cancer cell lines (Suppl. Table S1) were cultured in the presence or absence of tyrosine kinase inhibitors erlotinib or dasatinib. Cell proteins were extracted and analyzed by rosette and far-Western blotting using an array of SH2 domain probes (Suppl. Table S3). Bioinformatic analysis of quantified data was used for classification and biomarker screening.

We selected a group of 22 non-small cell lung cancer cell lines with known EGFR and K-RAS mutation status and known sensitivity to the EGFR TKI erlotinib (Suppl. Table S1). The overall strategy for our studies is shown in Fig. 1. Cell lysates were prepared from actively growing cells cultured in serum-supplemented medium. Two approaches to generate SH2 profiles were used, reverse-phase protein array and far-Western blotting [28]. In the first method, multiple protein samples (cell lysates) are spotted in arrays in register with the wells of a 96-well chamber apparatus. Each well is then filled with a solution containing a different GST-SH2 domain probe, and after incubation and washing, the bound probe is quantified for each spot. The amount of binding depends on the number and affinity of tyrosine phosphorylated protein sites in the sample. With this approach, which we term the “rosette” assay, it is possible to profile the total level of binding for virtually all SH2 domains in the genome (94 SH2 domain probes and one PTB domain representing 90 distinct proteins) using minimal amounts of protein sample.

**Figure 2:**
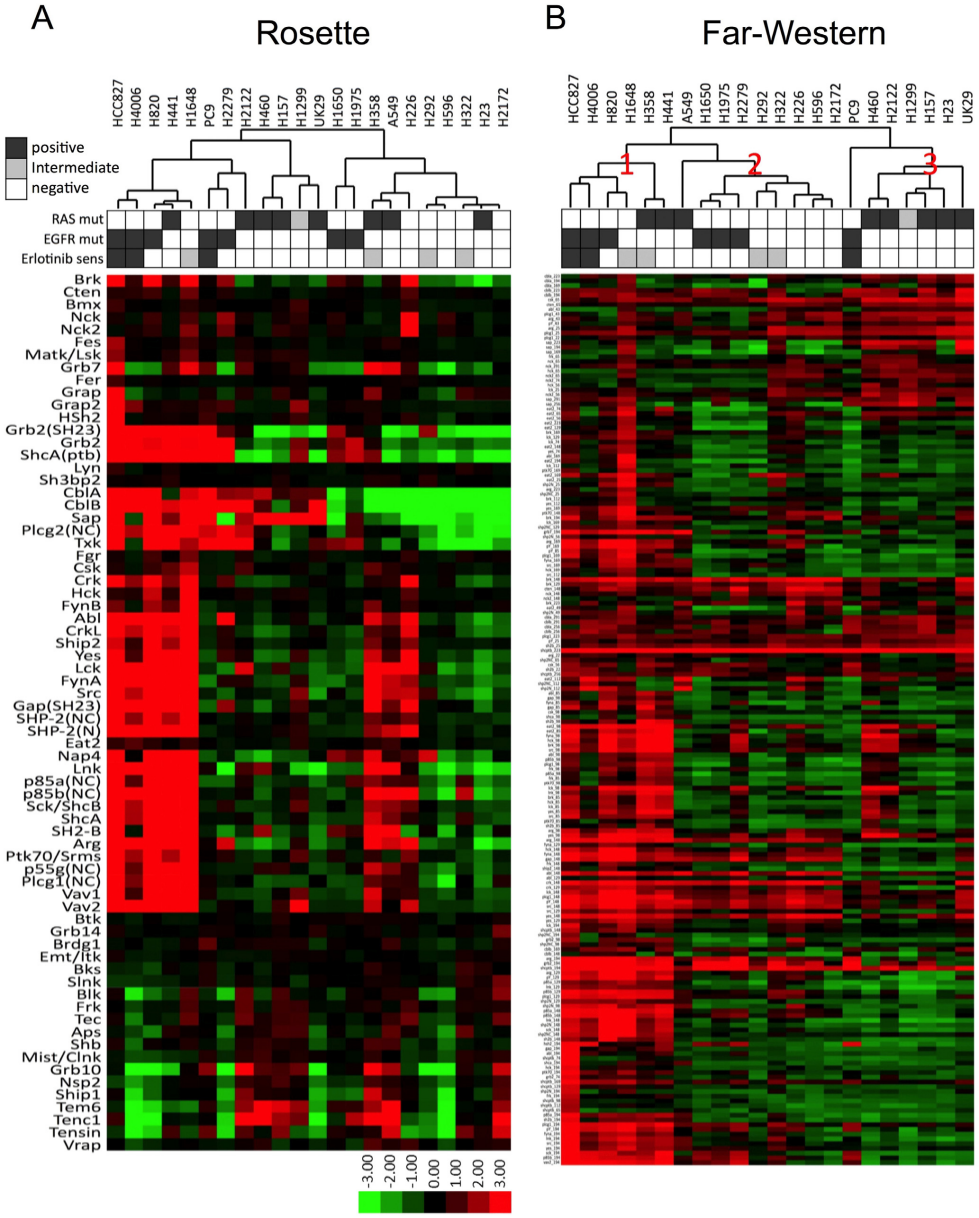
Unsupervised clustering of lung cancer cell lines based on SH2 binding. (**A**) Rosette data clustered by SH2 domain and cell line. Each row represents a single SH2 domain and each column represents a single cell line. Biological characteristics (EGFR mutation, K-RAS mutation, erlotinib sensitivity) are shown above in black and white. For erlotinib sensitivity, positive/sensitive: IC_50_ <10 nM; intermediate/moderately sensitive: 10–1000 nM; negative/insensitive: >1000 nM. (**B**) Far-Western data clustered by SH2 domain-specific bin and cell line. Each row represents a single MW bin (20 bins/lane) for a particular SH2 domain and each column represents a single cell line.

To investigate the relatedness of different cell lung cancer cell lines, quantitative SH2 binding values were subjected to unsupervised hierarchical clustering analysis (see Methods). Results are shown in heat map format in Fig. 2A and the raw image data is shown in Suppl. Fig. S1. Data with low signal/background were discarded; data for the remaining 70 probes were median-centered for clustering; red indicates higher than median binding, green lower. The processed data can be found in Suppl. Table S2. Data can also be accessed using a web-based viewer (http://proteome.moffitt.org/sh2/). In this analysis, cell lines harboring mutant EGFR cluster together in three distinct sub-clusters, while two large clusters (of four and eight cell lines) consist entirely of lines with wild-type (wt) EGFR. These results suggest that SH2 profiling can identify subsets of lung cancer cells, and that such clusters appear related to EGFR mutation status.

The second SH2 profiling approach uses far-Western blotting to obtain more detailed information about the relative abundance and apparent molecular weight (MW) of phosphoproteins that bind different SH2 domain probes. Protein samples are separated on the basis of size by gel electrophoresis and transferred to membranes, which are then probed with labeled SH2 domains. SH2 binding proteins are revealed as bands, and the apparent MW of these bands may provide clues to their identity. We developed software tools that allow SH2 binding data from far-Western blots to be quantified in “bins” by apparent MW, e.g. 20 bins per lane. The data from each bin (corresponding to phosphoproteins of a particular MW range that bind to the SH2 probe) can then be used as the basis for classification of samples. Thus instead of a single value for each sample and SH2 domain, as in the rosette assay, quantitative far-Western blotting provides at least 20 different data points, greatly increasing the potential discrimination between samples.

Far-Western blots of lung cancer cell lines were probed with 36 SH2 or PTB domains, as well as anti-pTyr antibody. Raw image data can be found in Suppl. Movie S1, and quantitative data in Suppl. Table S2. When quantitative results were subjected to hierarchical clustering, the samples clustered into 3 distinct classes, plus two outliers (Fig. 2B). One of these (cluster 3) consists entirely of cells with wt EGFR and is highly enriched for cells with activating K-RAS mutations. In contrast, clusters 1 and 2 are enriched for cells with EGFR mutations; within each of these clusters, cells with wt and mutant EGFR are segregated. Thus quantitative far-Western blotting appears to provide additional information on tyrosine kinase signaling state that can be used to functionally classify cells on the basis of RTK activation status. Overall the clustering results from the Rosette and far-Western assays are similar but not identical, as discussed below.

### Binding of a set of SH2 domains is enhanced in cells harboring activating EGFR mutations

**Figure 3:**
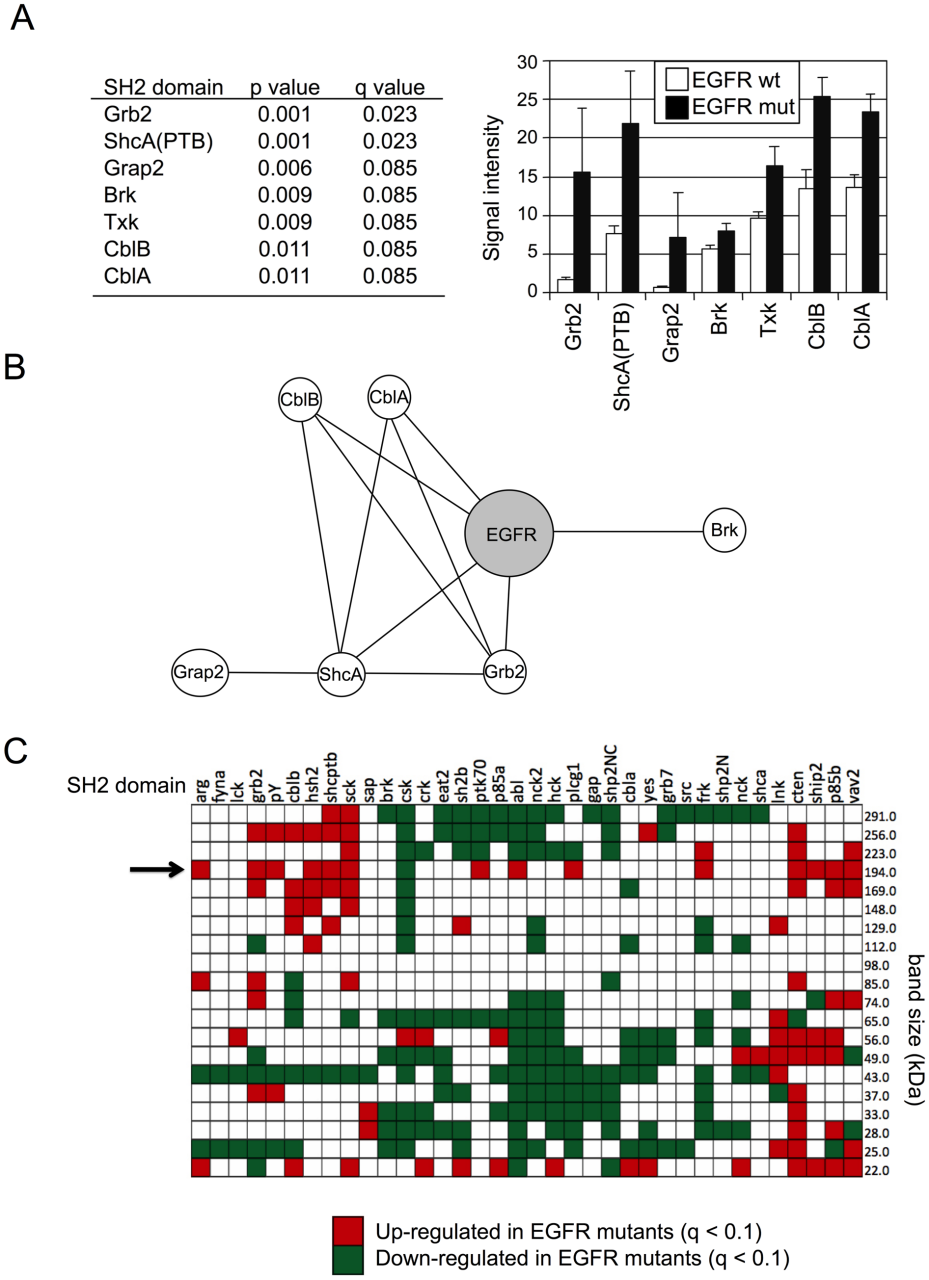
SH2 domains correlated with EGFR mutation. (**A**) SH2 domains whose binding is significantly correlated with EGFR mutation (q<0.1). Bar plot of SH2 signal for mutant and wild-type EGFR cell lines is shown as mean with standard error bars. "SH2 domain" is used in figures for all probes, including PTB domains and anti-pTyr antibody. (**B**) Protein-protein interaction map for EGFR (gray circle) and proteins with SH2 domains whose binding is significantly correlated with EGFR mutation (white circles). Lines indicate reported direct binding interactions. (**C**) Far-Western domain-specific bands significantly correlated with EGFR mutation. Colored boxes indicate the results of statistical significance in a Mann-Whitney test for differences (q<0.1) among the 22 cell lines shown in Figure 2B. Arrow indicates bin corresponding to EGFR family members. The numbers adjacent to bins indicate apparent MW, e.g. "291.0"  =  MW between 256–291 kDa.

We next asked whether the binding of any individual SH2 domain probes was highly associated with activating EGFR mutations. From rosette binding experiments we identified 7 probes whose binding was correlated in a statistically significant fashion with EGFR mutation status: Grb2, ShcA(ptb), Grap2, Brk, Txk, CblB and CblA (Fig. 3A) (refer to Suppl. Table S3 for SH2 domain names and corresponding proteins). We input these domains into PPI Spider, a tool for interpreting proteomics data in the context of known protein-protein interaction networks. This analysis showed that five of these proteins (ShcA, Grb2, CblA, CblB, and Brk) have been reported to bind directly to EGFR, while Grap2 is potentially linked to EGFR through ShcA (Fig. 3B). The fact that binding sites for Grb2 and ShcA (and the close Grb2 relative Grap2) are closely associated with EGFR mutation is particularly intriguing, as increased binding of these SH2 domains would be strongly predicted to lead to activation of the RAS signaling pathway via recruitment of the RAS activator Sos [32], [33].

A similar analysis was performed using data from far-Western blotting. We found that a number of specific bands on far-Western blots correlated significantly with EGFR mutation (Fig. 3C). Here it is interesting to consider bands whose binding tends to increase in EGFR mutant cells (red) versus those whose binding tends to decrease in EGFR mutant cells (green). We note that for probes predicted (on the basis of known signaling activity) to be associated with stimulation of the RAS pathway (Grb2 and Shc), increased binding in the molecular weight range containing phosphorylated EGFR family members (MW194, arrow) is seen for EGFR mutant cells. This likely reflects increased binding of these effectors to abnormally activated EGFR, compared with the wt receptor. This is also true for SH2 domains of other known positive effectors of EGFR signaling, including Vav2, PI 3-kinase (PI3K; P85B), and PLCγ Plcg1).

In contrast to EGFR mutation, we found no individual SH2 domains whose binding correlated strongly with RAS mutation status by rosette or far-Western blotting. Despite this, we noticed an interesting apparent correlation between clustering based on global SH2 profiles and K-RAS mutation status. This is particularly clear in the case of far-Western blotting (Fig. 2B), where two major clusters consist entirely of cells with wt K-RAS, whereas a third major cluster (cluster 3) consists almost entirely of cells with wt EGFR but mutant K-RAS. We were initially puzzled by the appearance of H1299 in cluster #3, as K-RAS is wt in these cells. However, further examination of sequence data revealed that the K-RAS relative N-RAS is mutated in H1299 (http://www.sanger.ac.uk/genetics/CGP/CellLines/), but not in any of the other cell lines used in this study. Thus a prediction based on SH2 profiling, that H1299 cells are likely to harbor activated RAS, was borne out, strongly validating the biological relevance of this approach. This cluster of RAS mutants (6 of 6 cell lines harboring RAS mutation) is highly unlikely to have occurred by chance (p = 0.001, permutation test, n = 100,000). These results indicate that tyrosine phosphorylation patterns can sub-classify cells based on RAS mutation status. The lack of correlation between individual SH2 domain probes and RAS mutation (as opposed to correlation based on the global tyrosine phosphorylation profile) may reflect the fact that RAS functions downstream of tyrosine kinases. We are currently testing the model that constitutive Ras activity leads to activation of feedback pathways that broadly downregulate tyrosine kinase signaling.

### A set of SH2 probes is correlated with sensitivity of lung cancer cells to EGFR TKI

**Figure 4:**
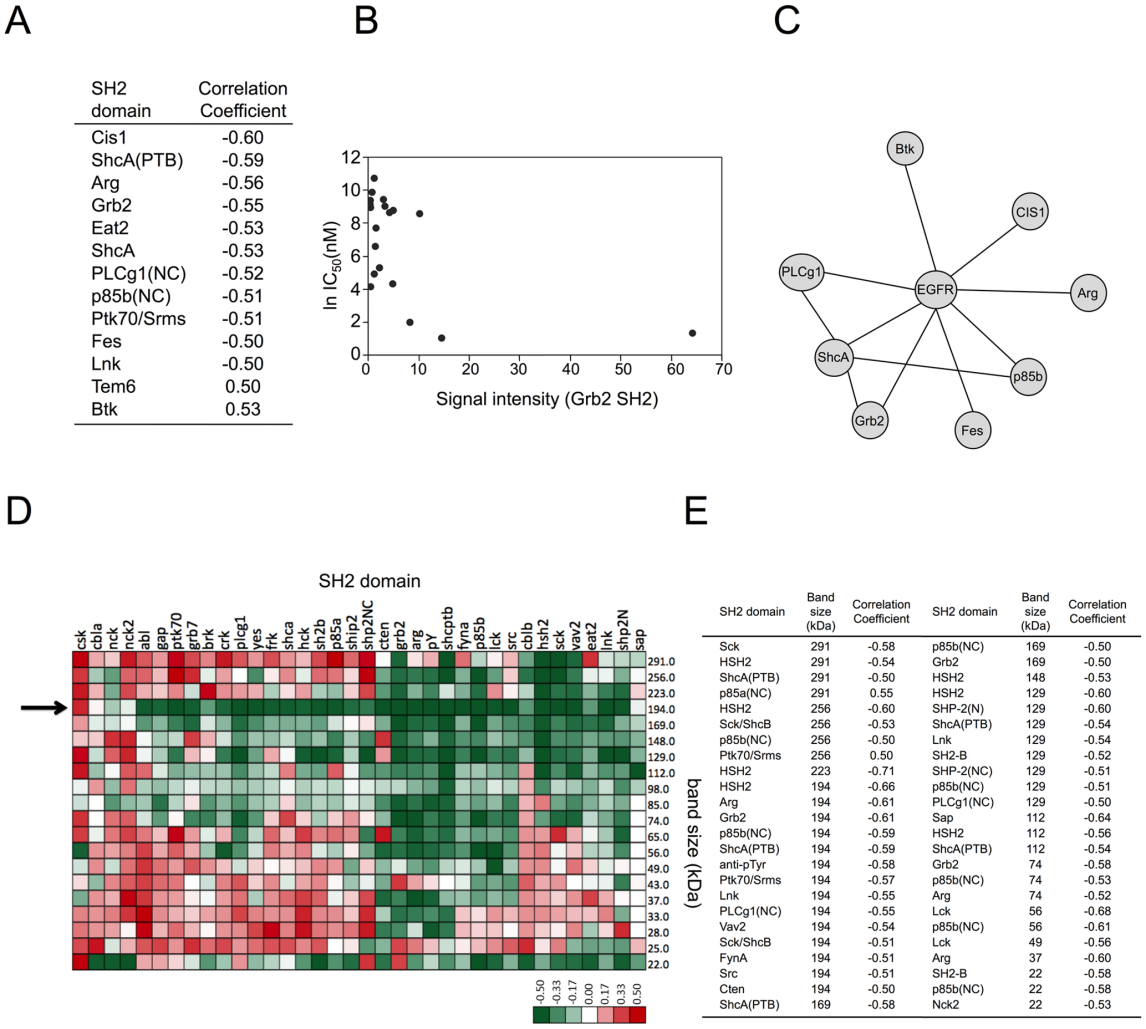
Correlation of SH2 binding with erlotinib sensitivity. SH2 domain signal in untreated cells was compared to ln IC_50_ for erlotinib.(**A**) Domains significantly correlated to ln IC_50_. Negative correlations indicate higher SH2 binding in cells more sensitive (lower IC_50_) to erlotinib. (**B**) Scatter plot of Grb2 SH2 domain binding vs. the ln (IC_50_) for erlotinib. (**C**) Protein-protein interaction map for EGFR and proteins with SH2 domains whose binding is significantly correlated with erlotinib sensitivity. Lines indicate reported direct binding interactions. (**D**) Heatmap representing correlation of each domain-specific bin to erlotinib sensitivity. Pearson's correlation was calculated for each domain-specific bin across 22 cell lines and the ln (IC_50_) for the corresponding cell line. Each heatmap location represents correlation coefficient for that bin; green indicates increasing SH2 signal with decreasing IC_50_ values (greater sensitivity); red indicates increasing SH2 signal with increasing IC_50_ values (see color bar). Arrow indicates MW bin containing EGFR family proteins. (**E**) List of the 46 domain-specific bins with |correlation coefficient| >0.5.

We next determined if SH2 domain binding correlated with the sensitivity of lung cancer cell lines to erlotinib, a small molecule EGFR kinase inhibitor. Such domains could serve as the basis for predictive biomarkers to identify tumors likely to respond to TKI therapy. SH2 profiling data were examined for possible correlation with the IC_50_ for erlotinib for each cell line (IC_50_ was assayed for this study under the same culture conditions used for SH2 profiling). We found that the binding of 13 probes corresponding to 12 proteins correlated in a statistically significant fashion with erlotinib IC_50_ (Fig. 4A). These include Grb2 (shown in Fig. 4B), ShcA, ShcA (ptb), p85B, Cis1, Arg, Eat2, Plcg1, Ptk70/Srms, Fes, Lnk, Tem6, and Btk. Network analysis confirms reports of direct interaction between EGFR and Grb2, ShcA, p85B, Cis1, Arg, Plcg1, Fes, and Btk (Fig. 4C). Overall the results are consistent with a model where erlotinib sensitivity is associated with particularly strong signaling from activated EGFR to known core downstream effectors, including the MAPK (Grb2 and ShcA), PI3K/Akt (p85B), and PLCγ (PLCg1) pathways.

Far-Western blotting also identified bands that correlated with erlotinib sensitivity (Fig. 4D&E). For a large number of SH2 domains, higher apparent binding to EGFR family members (arrow at MW194 in Fig. 4D) was associated with erlotinib sensitivity, while CSK (which downregulates Src-family kinases) was unique in that lower apparent binding of its SH2 domain to EGFR was associated with erlotinib sensitivity. These results were very similar to those seen for EGFR mutation (Fig. 3C), suggesting that increased binding of RAS activators and decreased binding of CSK to EGFR are associated both with EGFR mutation and erlotinib sensitivity. While in principle our analysis could be confounded by the known correlation between EGFR mutation and erlotinib sensitivity, we included in our analysis several lines that are resistant to erlotinib despite having activating EGFR mutations (H1975, H820, H2279, and H1650), as well as lines with wt EGFR that are sensitive to erlotinib (H292, H358, H1648, and H322). It is particularly intriguing that CSK is the only SH2 domain whose binding to the region of the blot containing EGFR decreases in both EGFR mutant cells and in cells sensitive to erlotinib. CSK negatively regulates Src family kinases, a key class of nonreceptor tyrosine kinases [34]. Although CSK SH2 binding is relatively weak and differences between the cell lines are modest, we have confirmed the statistical significance of the correlation in several separate experiments (Suppl. Fig. S2).

### MET activation is captured by SH2 profiling

**Figure 5:**
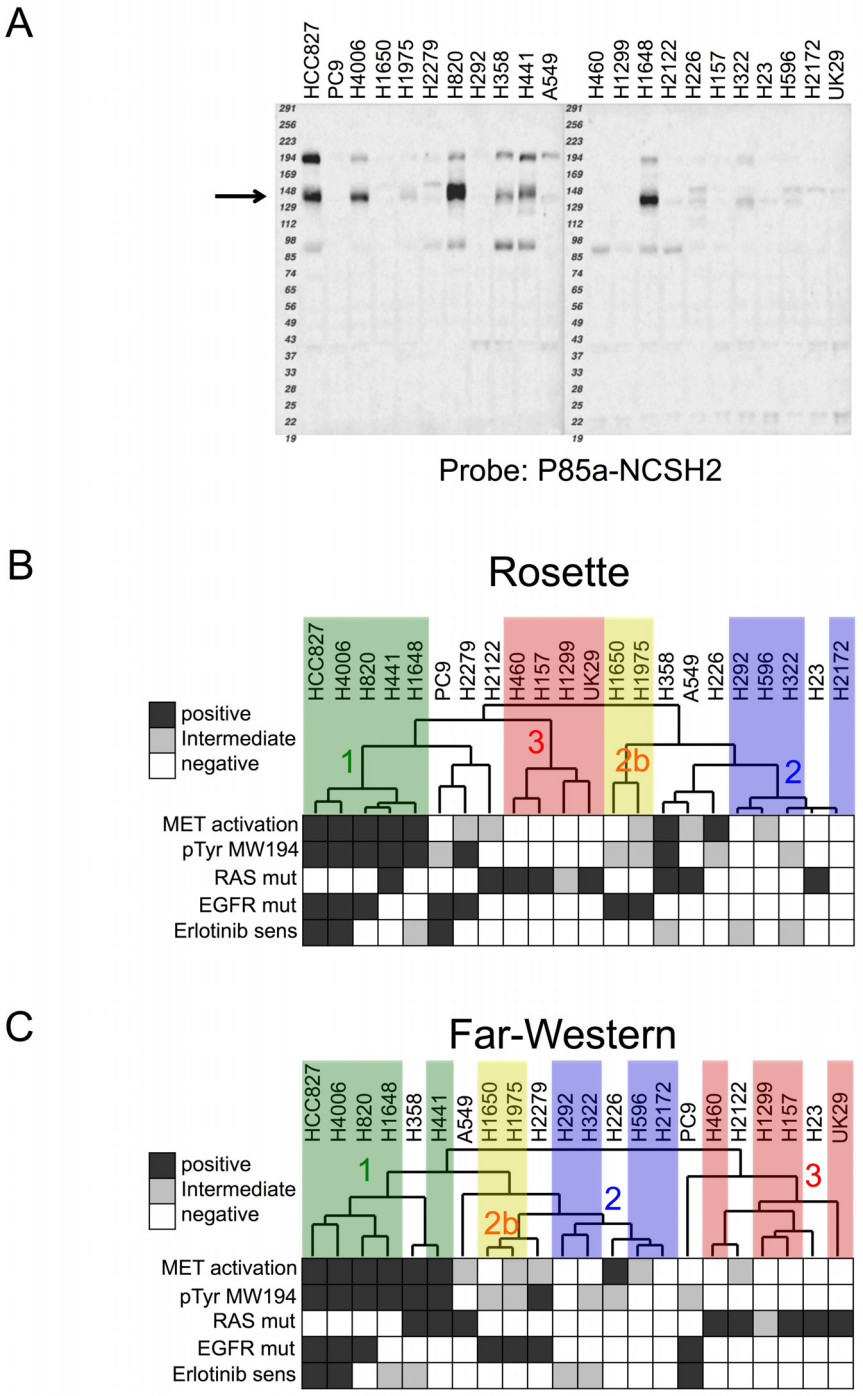
SH2 profiling identifies cells with EGFR and MET co-activation. (**A**) Far-Western blot of lung cancer cell lines probed with p85A SH2 domains. Note prominent band of ∼145 kDa in H820 (arrow). (**B,C**) Hierarchical clustering related to MET and EGFR family activation. Hierarchical clustering based on rosette data (B) or far-Western data (C), as previously shown in Fig. 2. Colors indicate cell lines that co-cluster by both rosette and far-Western analysis (clusters 1, 2, 2b, and 3). MET activation  =  immunoreactivity with phosphospecific MET antibody. pTyr MW 194 =  immunoreactivity with anti-pTyr in 194 kDa bin, which is a readout of EGFR family activation (see Suppl. Fig. S3). Cutoffs for MET activation: positive, >50% highest value in immunoblotting with anti-MET pY1334/1335; intermediate, 25–50%; negative, <25%. Cutoff for pTyr MW194: positive, >25% highest value in immunoblotting with anti-pTyr; intermediate, 10–25%; negative, <10%.

A potential strength of SH2 profiling would be to identify tumors in which multiple hyperactivated tyrosine kinases act in concert to drive downstream signaling, so we investigated whether SH2 profiling could identify cell lines in which MET and EGFR are co-activated. The MET kinase is amplified in H820 and H1648 cells [20], [35], which cluster closely together by both Rosette and far-western SH2 profiling (Fig. 2). In far-Western blots, we also noted strong binding of multiple SH2 domains including p85a to the region of ∼148 kDa in H820 and H1648. We observed a similar band at ∼148 kDa for a number of other cell lines, including HCC827, H4006, H358, and H441 (Fig. 5A). Suspecting this band was a marker for MET activation, we tested this by probing immunoblots with a phosphospecific antibody that specifically recognizes activated MET (Suppl. Fig. S3A). This analysis, together with immunoprecipitation and inhibitor studies, demonstrated that phosphorylation of the 148 kDa band was strongly dependent on MET activation (its presence correlated with activated MET and was inhibited by MET-specific TKI), but the band was not the MET receptor itself (Suppl. Fig. S3B,C). At the same time, we indirectly examined the activity of EGFR family members by quantifying tyrosine phosphorylation of the ∼194 kDa region of immunoblots, where EGFR family members are found (Suppl. Fig. S3A). Immunoprecipitation and inhibitor studies showed that most of the SH2 binding signal in this region of the blots could be attributed to EGFR family members (Suppl. Fig. S3B,C).

We then related MET and EGFR activation state with SH2 profiling results. Remarkably, unsupervised clustering of both rosette and far-Western data revealed that most cell lines with strong MET activation cluster in a distinct, tight group (Fig. 5B&C). Both by rosette and far-Western-based profiling, most cell lines with strong MET activation form a single cluster (cluster 1) that is clearly separated from the other cell lines. All of these cell lines also exhibit strong tyrosine phosphorylation of proteins co-migrating with EGFR, suggesting co-activation of MET and EGFR signaling in this group. Information on MET and EGFR activation also allowed us to more broadly compare clustering results based on rosette and far-Western data. This comparison revealed that four distinct clusters were common to both methods (Fig. 5B,C), encompassing 15 out of the 22 cell lines (7 lines cluster differently when the two methods are compared). Cluster 1 consists of cell lines with strong activation of both EGFR and MET signaling. Cluster 3 consists of cell lines with mutant RAS and low EGFR and MET activity, all of which are erlotinib resistant. Cluster 2 can be divided into two subgroups based on EGFR mutation status. From a clinical perspective these two subgroups are the most interesting: one consists of lines with mutant EGFR that are erlotinib resistant (cluster 2b), and the other includes several lines with wt EGFR that are erlotinib sensitive.

**Figure 6:**
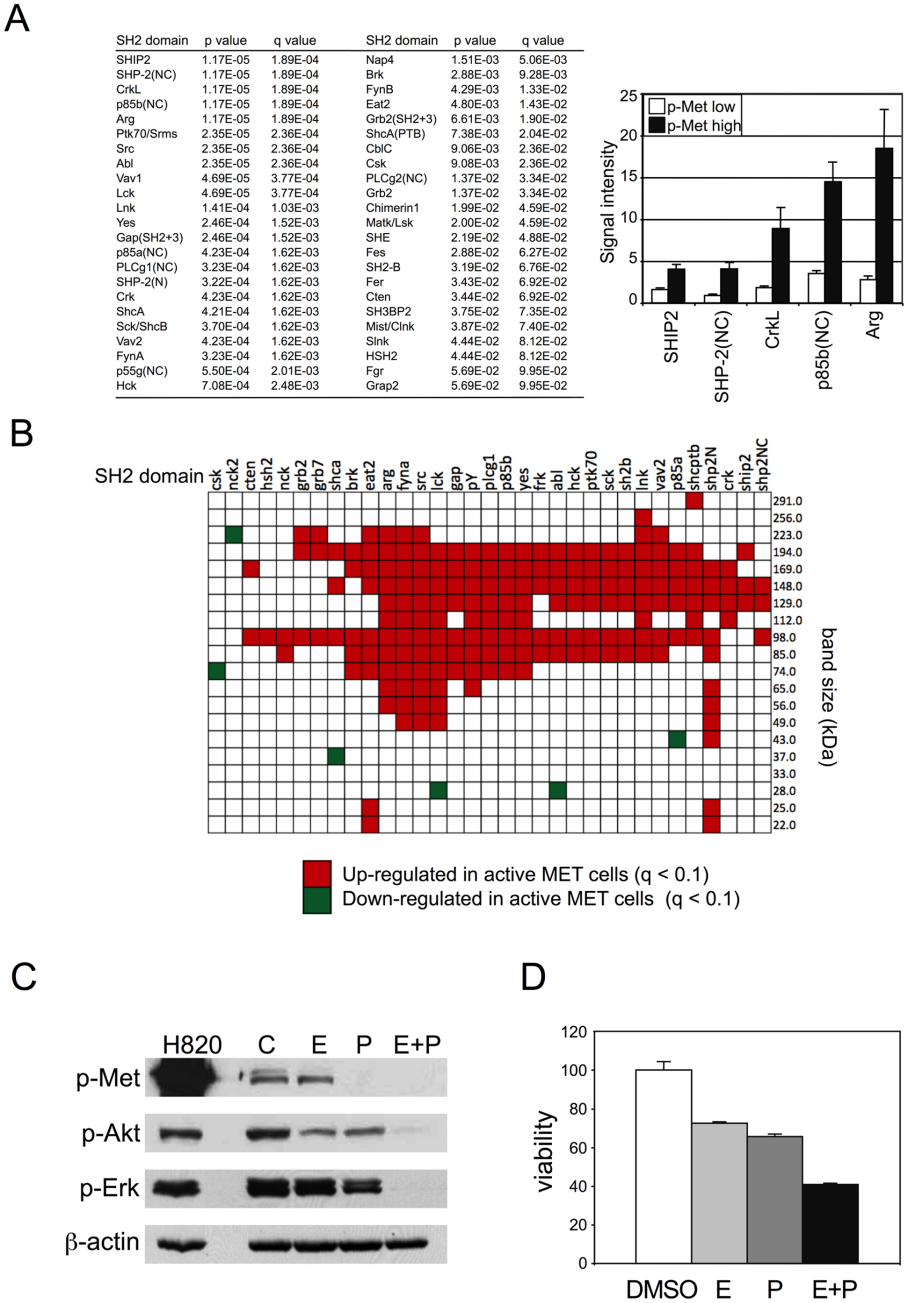
SH2 domains correlated with MET activation. (**A**) SH2 domains correlated with MET phosphorylation (p<0.01, q<0.1). Bar plot of SH2 signal for high and low MET phosphorylation. Mean and standard errors are shown. For this analysis “Low” includes both intermediate and low/negative categories (Fig. 5). (**B**) Far-Western domain-specific bands correlated with MET phosphorylation. Colored boxes indicate statistical significance in Mann-Whitney test for differences (q<0.1). (**C**) H1648 cells were exposed to control (DMSO), 1000 nM erlotinib (E), 1000 nM PHA665752 (P), or combination (E+P) for 3 h and analyzed by immunoblotting. Lysates from untreated H820 cells served as control for p-MET. Anti-β-actin was used to confirm equal loading. (**D**) Cell viability for H1648 cells exposed to 60 nM erlotinib (E), 300 nM PHA665752 (P), or combination (E+P).

Next we tested whether the binding of any individual SH2 domain probes was associated with MET activation. By rosette analysis we found that the binding of 46 SH2 domains was significantly associated with MET phosphorylation (Fig. 6A). Similarly, far-Western analysis showed a large number of bands associated with MET phosphorylation status (Fig. 6B). The number of SH2 domains and markers that correlate with MET activation is larger than those associated with EGFR mutation, suggesting MET activity has a more profound effect on overall pTyr patterns than EGFR mutation.

The fact that the H1648 cell line clustered tightly with the H820 cell line, which had previously been found to have an activating EGFR mutation along with MET amplification [20], suggested that H1648 cells may be similar to H820 in that downstream signaling is driven by both EGFR and MET. To test this, we exposed H1648 cells to inhibitors of EGFR (erlotinib), MET (PHA665752) [36], or the combination and examined downstream Akt and ERK activation (Fig. 6C). We observed modest reductions in phosphorylated Akt and ERK in response to either inhibitor alone, but strong inhibition upon dual EGFR and MET inhibition. The effects on cell viability mirrored the signaling responses, as the combination of both agents resulted in enhanced inhibition of cell growth (Fig. 6D). Thus global pTyr patterns, assayed by SH2 profiling, predict MET activation and may predict response to MET TKI in these cell lines.

### Perturbation of global pTyr profiles by TKIs

We next used SH2 domain profiling to investigate changes in global tyrosine phosphorylation in cells exposed to TKIs. Our expectation was that changes in SH2 binding patterns could be correlated with biological responses to inhibitors, and that probes for which binding decreased strongly in TKI-inhibited cells were likely to represent key pathways for TKI action. Four lung cancer cell lines (H292, H441, H358 and HCC827) were briefly exposed to erlotinib, an inhibitor of EGFR, or dasatinib, a SRC family kinase inhibitor that inhibits multiple tyrosine and serine/threonine kinases [37], [38], [39]. These cell lines differ greatly in their responses to these TKI. In HCC827 cells with activating EGFR mutation, both inhibitors induce apoptosis; in H358 and H292 cells with wt EGFR, both agents induce cell cycle arrest; and H441 cells with wt EGFR are resistant to both agents ([9] and data not shown). Rosette and far-western SH2 profiling was performed for all four cell lines in both the treated and untreated groups.

**Figure 7:**
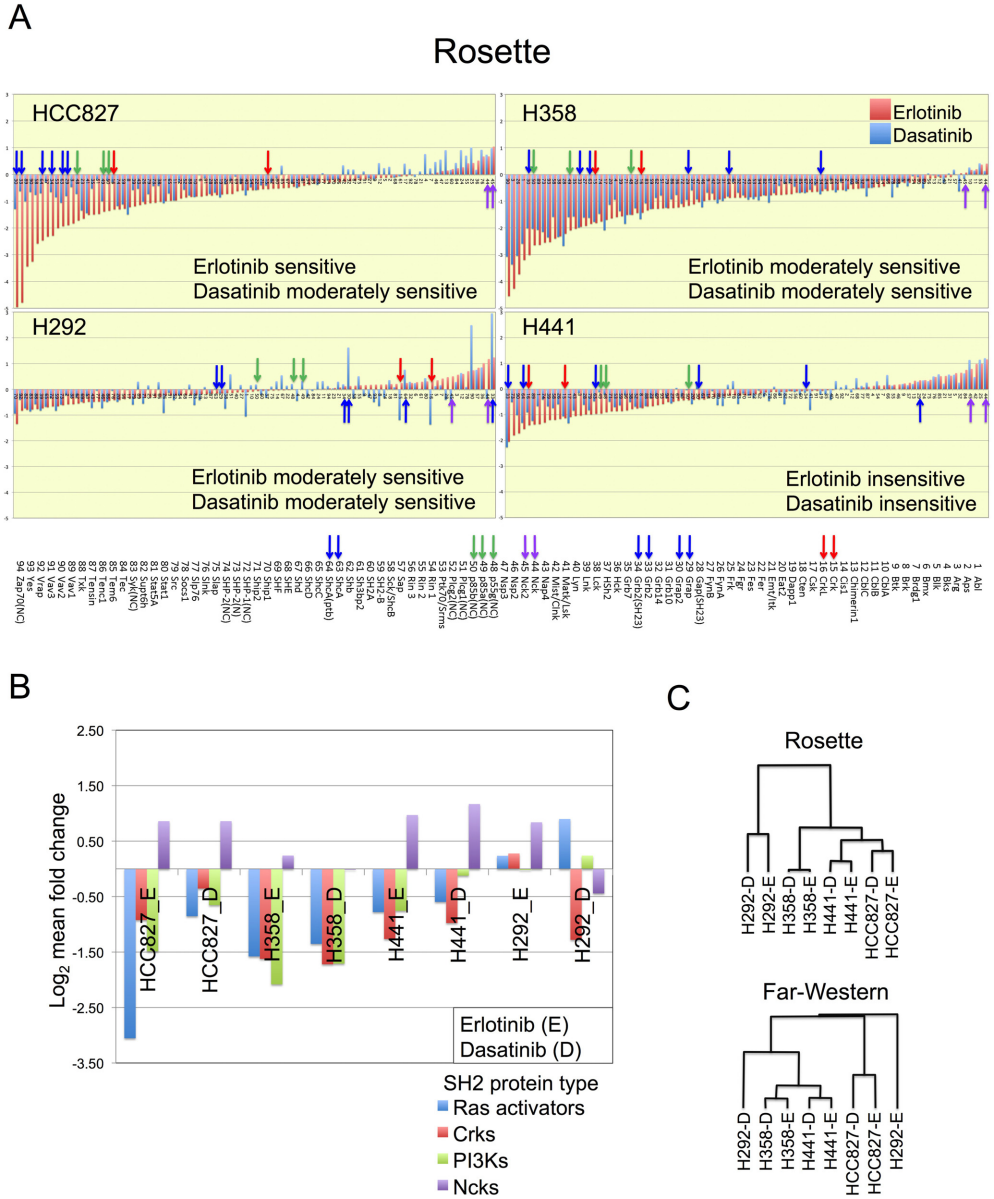
SH2 profiling of cells treated with erlotinib or dasatinib. Change in SH2 binding in presence of EGFR TKI (erlotinib) or SRC TKI (dasatinib) for four lung cancer cell lines (H292, H358, H441 and HCC827). Cells were treated for 1 h with inhibitors prior to analysis. (**A**) The log_2_ fold change in SH2 binding upon erlotinib and dasatinib treatment (relative to untreated) as determined by rosette assay is shown for all probes, ranked in order of fold change in response to erlotinib. Each cell line is represented in a separate panel. For each SH2 domain probe, values for changes upon erlotinib treatment are shown as red bars, for dasatinib as blue bars. Colored arrows indicate position of SH2 domains for RAS activators (blue), PI3Ks (green), Crk family (red), and Nck family (purple). (**B**) Bar graph of mean log_2_ fold change for indicated SH2 domain groups and cell lines treated with erlotinib (E) or dasatinib (D). (**C**) Dendrograms obtained from hierarchical clustering of log_2_ fold changes (TKI-treated vs. untreated).Results for rosette assay are shown on top and far-Western blotting below. See Suppl. Fig. S5A for heatmaps.

Rosette binding data (log_2_ fold changes in treated versus untreated groups) were visualized in waterfall plots ranking changes in SH2 binding (Fig. 7A, Suppl. Fig. S4A). In HCC827, erlotinib caused complete collapse of pTyr signaling, as large decreases in SH2 binding are observed for almost all probes. The most significant reductions in binding occurred for the Grb2, Grap2, Vav2, and Vav1 SH2 domains. Broadly similar changes were observed upon dasatinib treatment, suggesting an overlap of mechanism, yet fold changes were less for most probes, consistent with its less potent effects on EGFR phosphorylation [9]. Similar to HCC827, binding of almost all SH2 domain probes was markedly reduced in TKI-treated H358 cells, and there was even greater similarity between the responses to erlotinib and dasatinib. In H441, which is resistant to TKI, erlotinib and dasatinib evoke similar overall patterns of change as H358. However the decrease in SH2 binding was blunted in H441 compared to H358 and HCC827, and the binding of a larger number of SH2 domains increased in the presence of both inhibitors compared to untreated cells. Finally, H292 showed the least dramatic changes in SH2 domain binding, and the overall pattern of response to TKI treatment in this cell line was very different from the other three. Furthermore, the erlotinib and dasatinib profiles were more dissimilar compared to the other cell lines tested. The same samples were also analyzed by far-Western blotting using a limited set of SH2 probes (Suppl. Fig. S4B). These data showed that responses to erlotinib and dasatinib were quite similar in the H358 and H441 cells, again arguing that the two agents have similar targets in these cells.

In an effort to understand the biological implications of these results, we compared the response of several classes of SH2 domains to TKI treatment. We chose Grb2 and Shc family members (Grb2, Grb2(SH23), Grap, Grap2, ShcA, ShcA(ptb)) because of the known importance of Ras activation to tumorigenesis, and our earlier results linking Ras activation to erlotinib sensitivity (Fig. 4); and PI3Ks (p85A, P85B, P55G), because of the strong association between PI3K/Akt activity and cancer [40]. We also analyzed binding of Crk proteins (Crk and CRKL) and Nck proteins (Nck1 and Nck2), based on the involvement of these adaptors in RTK-dependent actin cytoskeletal rearrangements [41], [42] and our unpublished observations that these SH2 domains bind to distinct targets compared to most RTK effectors. We found that binding of the SH2 domains of RAS activators, Crks, and PI3Ks decreased with TKI treatment, with the exception of H292 cells (Fig. 7B). Ncks were unique in that SH2 binding consistently increased upon TKI treatment (Fig. 7B, Suppl. Fig. S4B). This is consistent with other work showing that Nck binding sites diminish upon RTK stimulation, in concert with loss of focal adhesions and stress fibers ([42] and L. Jia, KM, and BJM, unpublished observation). On average, the degree of change in binding of RAS activators approximates the erlotinib sensitivity of the cells; for HCC827, H358, and H441 cells the average log_2_ fold change is −3.1, −1.6, and −0.8, respectively. For dasatinib, inhibition of the PI3K pathway may also be important, as PI3K SH2 binding is reduced significantly in H358, whereas in the resistant H441 line binding is virtually unchanged. H292 cells have an anomalous pTyr response to TKI that may reflect very low overall tyrosine phosphorylation levels and lack of either EGFR or KRAS mutation in this line.

When quantitative changes in SH2 binding upon TKI treatment were used as the basis for unsupervised hierarchical clustering, several general principles become apparent (Fig. 7C, Suppl. Fig. S5). For HCC827, H358, and H441, the same cell lines treated with different inhibitors were more similar in their responses than different cell lines treated with the same inhibitor. Thus for each of these three cell lines, the critical molecular targets for both erlotinib and dasatinib are likely to overlap. However, the dissimilarity in the responses to TKI when different cell lines are compared indicates that these critical targets are not identical in the three lines. The fact that the response of a particular cell line to different TKI is more similar than the response of different cell lines to the same TKI is interesting and rather unexpected, suggesting that TKI response is highly dependent on the cell-specific signaling milieu. This idea is reinforced by the anomalous response of H292, which suggests these cells are driven by very different molecular abnormalities, and that their TKI-induced cell cycle arrest is mechanistically distinct from that seen in the other lines. Therefore SH2 profiling provides novel insight into the mechanisms whereby TKI suppress tumor cell growth, though much more work is needed to correlate particular phosphorylation changes with biological responses to TKI.

## Discussion

Our results strongly suggest that SH2 profiling is a useful molecular diagnostic approach for analyzing tyrosine kinase signaling in tumor cells. Not only can the overall SH2 profiling patterns serve as the basis for classification of tumors with potential prognostic and/or predictive value, but this approach can also identify molecular probes and phosphorylated proteins that may individually or in combination serve as clinically useful biomarkers. This strategy combines the advantages of global analysis, which does not depend on preconceived notions of the key molecular drivers involved, with a tight focus on tyrosine phosphorylation, which is known to play a central role in many aspects of tumor biology.

Our studies were initially driven by the hypothesis that in lung cancer cell lines, tyrosine phosphorylation patterns would be related to the state of EGFR signaling, and in particular to EGFR mutation status. Consistent with this idea, we found higher binding of several SH2 probes, including Grb2 and ShcA, in EGFR mutant cells. However mutant EGFR does not drive the entire tyrosine phosphorylation pattern in lung cancer, as cells with mutant EGFR can form distinct clusters. This is perhaps not surprising given the abundance of tyrosine kinases implicated in lung cancer biology [4]. Our results are also consistent with the finding that distinct mutant alleles of EGFR can lead to measurable differences in tyrosine phosphorylated peptides [25]. SH2 profiling therefore reveals heterogeneity of downstream signaling outputs despite common genomic properties (EGFR mutation), and could therefore provide additional predictive or prognostic information in tumor classification.

It is interesting to note that classification based on SH2 profiles correlated quite closely with two other molecular markers, MET activation and K-RAS activation. In the case of MET, both rosette and far-Western SH2 profiling clearly distinguished a cluster of cells with high levels of activating MET phosphorylation. This strongly suggests that in lung cancer, global patterns of tyrosine phosphorylation are at least as dependent on MET activation as they are on EGFR mutation. This important and rather unexpected insight is a clear indication of the value of unbiased, global approaches.

The correlation we observed between SH2 binding pattern and activating RAS mutation (Fig. 2B) is also surprising, given that RAS is expected to act downstream of tyrosine kinases (thus constitutive RAS activation should render cells independent of pTyr-based signals). This result implies that SH2 profiling provides useful information even for tumors not driven by tyrosine kinase activation. Notably, of the three K-RAS mutant cells not included in the major cluster of K-RAS mutants (Fig. 2B), H441 and H358 have recently been reported to have a more epithelial phenotype [43], while A549 was shown to have a mixed epithelial-mesenchymal phenotype [44]. Thus, SH2 profiles may also reflect the epithelial phenotype in the context of K-RAS mutation. The fact that most K-RAS mutant cells cluster together also implies a common pattern of tyrosine phosphorylation in cells where RAS activity is decoupled from RTK activation. Such a pattern could be generated by feedback loops that normally function to downregulate pTyr signaling following RAS activation. We note that while overall SH2 binding is decreased in the mutant RAS cluster compared to other cell lines (see cluster 3, Fig. 2B), in far-Western blots the binding of the ubiquitin ligases CblA and CblB to high-molecular weight phosphoproteins is increased in these cells, consistent with Cbl-dependent downregulation of RTKs. Guha and colleagues also observed decreased tyrosine phosphorylation of several proteins in human bronchial epithelial cells expressing mutant K-RAS [25].

The results presented here suggest that SH2 domain binding sites could be used as biomarkers of EGFR TKI sensitivity. We found 13 probes whose binding correlated with erlotinib sensitivity. A number of these, including Grb2 and ShcA, are from proteins predicted to promote downstream activation of the RAS/MAPK pathway when bound to activated RTKs. Similar correlations were seen for SH2 domains of PI3K and PLCγ, two other key pathways downstream of EGFR signaling. It is particularly striking that binding sites for known RAS pathway activators are associated both with activating EGFR mutations and with erlotinib sensitivity. RAS activation has long been associated with proliferative signals, and RAS itself is activated by mutation in a large number of human cancers. By examining pTyr signaling in an unbiased fashion on a global scale, our results confirm the central importance of RAS signaling in lung cancer, and suggest that particularly strong activation of RAS may be a hallmark of cancers that are driven by EGF receptor mutation and are sensitive to EGFR inhibitors (i.e. are “addicted” to EGFR signaling). It will be important to determine how SH2 profiling could add additional information beyond known predictors of EGFR TKI response such as EGFR mutation status, gene amplification, gene expression profiles, and autocrine signaling [13], [14], [15], [17], [18], [19], [45]. In particular, developing predictors based on multiple SH2 domain probes is likely to improve the discriminating power of SH2 profiling, and is therefore a high priority for future studies.

Importantly, SH2 domain profiling interrogates the entire spectrum of tyrosine phosphorylation sites, thereby detecting signaling driven by diverse tyrosine kinases in their native cellular environment. Using this approach we were able to identify a pattern of SH2 binding characteristic of cells driven both by EGFR and MET that could require dual EGFR and MET inhibition to block downstream signaling. In one example, we showed that in H1648 cells, which cluster together with other cell lines in which EGFR and MET signaling is co-activated, downstream signaling and cell growth were synergistically inhibited by erlotinib and PHA665752, a MET-specific TKI. Thus, information provided by SH2 profiling may be useful for guiding therapeutic decisions. Future studies will explore the potential correlation of SH2 binding patterns with activation of other RTKs implicated in lung cancer, such as IGFR, FGFR, PDGFR, ALK, and EPH receptors.

SH2 profiling is highly complementary to other current methods for analyzing global tyrosine phosphorylation patterns, such as phosphospecific antibodies and MS [46], [47]. Phosphospecific antibodies (raised against specific tyrosine phosphorylated sites) can be sensitive and specific, but have the disadvantage that they require knowledge of the relevant phosphorylated sites, and they are available for only a small fraction of known sites. Mass spectrometry (with or without initial enrichment by pull-down or immunoprecipitation using anti-pTyr antibodies) can be used to identify specific phosphorylated sites in a sample [4], [25], but has the disadvantages that coverage and sensitivity are modest, relatively large amounts of sample are required, and absolute quantification of individual sites is difficult. By contrast, SH2 profiling is comprehensive, highly sensitive, and quantitative, but has the disadvantage that the individual phosphoproteins responsible for SH2 binding are not identified. Ultimately, the combination of SH2 profiling with MS analysis of selected samples should allow the development of specific and sensitive molecular tests to discriminate tumor subtypes based on tyrosine phosphorylation pattern. We note that three cell lines used in this study (HCC827, H441, and H358) were also analyzed by Rikova et al. using MS [4]. Their clustering results (based on MS) and ours based on SH2 profiling agree that HCC827 and H441 are very closely related by pTyr pattern; in the case of H358, clustering based on MS and rosette suggest these cells are rather distantly related to the other two lines, while clustering based on FW blotting suggests a closer relationship.

Going forward, it will be important to demonstrate that primary human lung cancer samples exhibit SH2 binding patterns similar to those seen in lung cancer cell lines. Using the current rosette assay, it is possible to profile the level of binding for virtually all SH2 domains using less than 100 µg total protein. We and colleagues have also developed a high-throughput multiplexed SH2 profiling platform based on tagging of SH2 domains with specific oligonucleotides [27]. Because a PCR amplification step makes this assay extremely sensitive, it may provide the basis in the future for standardized clinical SH2 profiling assays for molecular diagnostics.

SH2 domain profiling of TKI-treated cells also provides a novel, global approach to understand kinase inhibitor activity in vivo. Comprehensive analysis of changes in tyrosine phosphorylation in response to TKI is likely to highlight key targets of inhibitor action and uncover differences in cellular responses to inhibitors. Surprisingly, we found some tumor cells (H358 and H441) had very similar pTyr responses to two distinct TKI (erlotinib and dasatinib), despite the different spectrum of kinases inhibited by these agents. In principle SH2 profiling could be used to compare multiple TKI responses and to group kinase inhibitors according to mechanism of action. This is analogous to the efforts of the Connectivity Project, which uses gene expression as a way to group chemical compounds with similar mechanism of action [48]. Given the promiscuity of some inhibitors such as dasatinib, this may be one way to assess the overall impact of inhibiting multiple kinases in concert [38], [39].

In conclusion, our results demonstrate that SH2 profiling can recognize distinct patterns of EGFR signaling in lung cancer cells, and more broadly provides a straightforward and comprehensive approach to profile the global state of tyrosine phosphorylation signaling in tumors. These results could inform therapeutic decisions regarding TKI in lung cancer, and can provide a novel basis for tumor classification complementary to existing methods. This approach also provides new insights into the basic wiring of tyrosine kinase signaling networks in tumor cells, and how those networks are affected by TKI treatment.

## Materials and Methods

### Cell Lines and Reagents

Human lung cancer cell lines H292, H358, H441, A549, H460, H1703 and H1299 were obtained from ATCC (Manassas, VA). HCC827 cells were provided by Dr. Jon Kurie (MD Anderson Cancer Center), H1648, H2122, H226 and H157 cells were provided by Dr. John Minna (UT Southwestern Medical Center), H322 were provided by Dr. Paul Bunn (University of Colorado), H23 cells were provided by Dr. Gerold Bepler (Moffitt Cancer Center), and UK29 cells were provided by Dr. Penni Black (University of Kentucky). All cell lines were maintained in RPMI-1640 medium supplemented with 10% newborn calf serum (NCS) from Sigma (St. Louis, MO). Erlotinib was provided by OSI Pharmaceuticals (Melville, NY), dasatinib by Bristol Myers Oncology (Princeton, NJ), and PHA665752 by Pfizer (San Diego, CA) [36]. Stock solutions in 100% DMSO were diluted directly in the media to indicated concentrations.

### SH2 Profiling

SH2/PTB domain binding assays were performed as described [28], [49]. Briefly, lung cancer cell lines were lysed in KLB buffer (150 mM NaCl, 25 mM Tris-HCl pH 7.4, 5 mM ethylene diamine tetraacetic acid (EDTA), 1 mM phenyl methyl sulfonyl fluoride (PMSF), 1% Triton X-100, 10% glycerol, 0.1% sodium pyrophosphate, 10 mM β-glycerophosphate, 10 mM NaF, 5 µg/ml of Aprotinin (Sigma A6279, St. Louis MO), 50 µM pervanadate). Precipitated proteins were resuspended in spotting solution (180 mM Tris–HCl, pH 6.8, 30% glycerol, 6% sodium dodecyl sulfate (SDS), 15% β-mercaptoethanol, and 0.03% bromophenol blue) and briefly boiled. 0.1 µl lysate (400 ng total protein) was spotted in arrays on nitrocellulose membranes and incubated with purified GST-SH2 or -PTB domains (∼100 nM) for 2 h. Probe binding was detected by enhanced chemiluminescence (ECL) (PerkinElmer, Waltham MA) using HRP-labeled anti-GST antibody (Sigma A7340) or GSH-glutathione conjugate (Sigma G6400) and digitally captured (Kodak Image Station). The binding assay was performed four times, including at least two separate experiments, and average signal intensity for each spot was quantified by densitometry (ImageJ v1.40).

Far-Western analysis for lung cancer cell lysates was performed as described [50], [51]. Briefly, proteins were separated by SDS-PAGE using NuPAGE precast gels (Invitrogen, Grand City NY) and transferred to nitrocellulose membranes. Replica membranes were incubated with GST-SH2 domains for 2 h, and bands were detected by ECL and digitally captured. Blots were stripped and reprobed several times with additional SH2 domains and anti-pTyr antibody (PY100, Cell Signaling, Beverly MA). To quantify bands on multiple blots derived from different gels, SH2 blot images were aligned in reference to corresponding anti-pTyr blots using Photoshop (Adobe). The aligned blot images from two independent experiments were quantified using a custom-made plug-in written for ImageJ densitometry (H. Zhang, J. Maddox, and D.G. Shin, University of Connecticut).

### Protein Expression Analysis

Cells were washed with ice-cold PBS and extracted with chilled lysis buffer (10 mM Tris, pH 8.0, 60 mM KCl, 1 mM EDTA, 1 mM DTT, 0.5% NP-40, 10 mM Na_3_VO_4_, 50 mM NaF, 1 mM PMSF, 1 µg/ml aprotinin, 1 µg/ml leupeptin, 1 µg/ml pepstatin). Total cellular proteins were separated on SDS-PAGE, transferred to nitrocellulose membranes, and probed with rabbit polyclonal antibodies specific for pTyr 1344/45 MET, pThr202/Tyr204-p44/42 ERK, and pSer473 AKT (Cell Signaling) and β-actin (Sigma). Detection was by horseradish-peroxidase conjugated secondary antibodies and ECL (Amersham, Piscataway, NJ).

### Drug Sensitivity Assays

Cell viability assays (MTT) were performed using the Cell Proliferation Kit (Roche, Indianapolis, IN) following the manufacturer's recommendations. Cells were plated at 2–5×10^3^ cells per well in 96-well plates, incubated overnight, exposed to a serial dilution of dasatinb or erlotinib in complete media with 5% newborn calf serum, and viability assessed after 5 d. The IC_50_ was calculated by non-linear regression analyses using MATLAB scripts that pair data points with sigmoidal curves that predict a signal response based on a four-parameter fit. Data presented represent three separate experiments with 8 data points separating each dose per condition. Data are expressed as mean ± SD.

### EGFR and K-RAS Genotyping

Genomic DNA extraction from each NSCLC cultured cell lines was performed using DNeasy Kit (Qiagen, Düsseldorf). Sequencing of exon 19, 20, and 21 of EGFR was performed as previously described [15]. For K-RAS, the primers were K-RAS exon 1 (forward), 5′ TTAACCTTATGTGTGACATGTTCTAA-3′ and (reverse) 5′-AGAATGGTCCTGCACCAGTAA-3′, which generates a fragment of 225 bp, and K-RAS exon 2 (forward), 5′-TCAAGTCCTTTGCCCATTTT-3′ and (reverse) 5′-TGCATGGCATTAGCAAAGAC-3′, which generates a fragment of 374 bp.

### Network Analysis

SH2 domain binding data were input into PPI Spider (http://mips.helmholtz-muenchen.de/proj/ppispider/). Analyses were run with 100 random networks and only proteins that directly connect to each other through no more than one edge were allowed for visualization. Networks were subsequently input into Cytoscape for visualization.

### Clustering Analysis

For rosette data, hierarchical clustering was performed on the 70 probes (after median centering) using full linkage and uncentered correlation using Cluster 3.0 and Java Treeview. For far-Western data, 720 domain-specific bands (20 bands×35 SH2 domains) were filtered to retain those with a standard deviation >5, and at least 11 of 22 cell lines having intensity above 5.0 for a specific domain-specific band. Hierarchical clustering was performed on the remaining 188 bands using full linkage and uncentered correlation using Cluster 3.0 and visualized using Java Treeview. No normalization was used in clustering, but for visualization purposes, the intensities were median-centered so that green represents values below the median probe value and red above. Further details on rosette and far-western data analysis are provided in the Suppl. Methods S1 and Suppl. Figures S6 and S7.

### Statistical Analysis

Details are described in Suppl. Methods S1. Briefly, hierarchical clustering was performed using Cluster 3.0 and Java Treeview. To identify probability of a clustering pattern occurring by chance, permutation tests were performed. Domains were identified as statistically significant with respect to dichotomous characteristics using a Mann-Whitney test applied to each domain. To correct for multiple testing problems, false discovery rates (q values) were calculated using the Q Value package in Bioconductor [52] and a 10% FDR (q≤0.1) was considered significant. Bar graphs showing differences between groups were displayed using mean and standard errors. Pearson's correlation coefficient was computed for each domain related to erlotinib sensitivity. To characterize changes with treatment, differences in signal were quantified as the log_2_ fold change (log_2_ (treated/untreated)) and hierarchical clustering was performed using these values.

## Supporting Information

Supplemental Methods S1 Supplemental methods and references.
(0.05 MB DOC)

Table S1 Characteristics of cell lines used in this study. Adeno  =  adenocarcinoma; Squamous  =  squamous cell carcinoma; BAC  =  bronchiolioalveolar carcinoma; Large  =  large cell carcinoma; NOS  =  not otherwise specified; WT  =  wildtype.
(0.06 MB DOC)

Table S2 Processed SH2 binding data. Sheet 1: raw rosette data used for the analysis. The matrix consists of 70 (SH2 domain probes)×22 (lung cancer cell lines). 26 domains with lower signal were removed based on statistical analysis (Figure S2; see also supplementary methods, below). Sheet 2: far-Western data used for the analysis. The matrix consists of 720 (SH2 domain probe_bin number) ×22 (lung cancer cell lines).
(0.36 MB XLS)

Table S3 List of probes used in this study. 96 far-Western probes consist of 94 GST-SH2 domains, a GST-PTB domain (ShcA), and GST alone (negative control). Probe name, protein name, Entrez GeneID, source organism, SH2 insert amino acid region, and structure of construct are shown.
(0.02 MB XLS)

Figure S1 SH2 rosette assays. The SH2 rosette assay (high throughput dot blotting) was performed as described [1], [2]. Lung cancer and control cell lysates were spotted in duplicate on nitrocellulose membrane as indicated (top) in register with wells of a 96-well chamber plate. Each well was separately incubated with GST-SH2/PTB domain, GST control, anti-phosphotyrosine antibody, or anti-actin antibody (probe list is on the right). Control  =  negative control (phosphatase-treated lysate); p-control  =  positive control (mixed lysates from pervanadate-treated cell lines); see [2] for details. Two independent experiments were performed in duplicate, providing four quantifiable data points for each probe/sample pair. Array images were background-subtracted and the integrated density of each spot was measured using ImageJ densitometry (v1.40).
(5.97 MB TIF)

Figure S2 Correlation of Csk SH2 binding and EGFR mutation status. Top: Signal intensity from far-Western analysis using CSK SH2 probe was quantified in multiple blots. Black bars represent average of two independent blots (Expt. 1; these data are provided in Suppl. Table S2 and were used for clustering analysis). White bars represent data from an independent blot performed at another time with a subset of cell lines. EGFR mutation status of cell lines (mut  =  mutant, wt  =  wild-type) is indicated below. Bottom: Average CSK signal intensity for mutant vs. wt cell lines. Differences are statistically significant by unpaired T test as indicated by P values.
(1.64 MB TIF)

Figure S3 MET and EGFR family activation. (A) Left: Lysates from lung cancer cell lines were separated by SDS-PAGE, transferred to membranes, and blotted with phosphospecific anti-MET antibody (recognizing pY1334/1335). Signal was detected by chemiluminescence and the pMET band (arrow) was quantified using ImageJ densitometry. Representative blot (upper) and quantified result from multiple experiments (lower) are shown. Right: Similarly, protein bands of approximately 194 kDa on anti-pTyr blots (at molecular weight of EGFR family members, arrow) were quantified. Values are given as percent of maximum signal. (B) Cell lines indicated were treated with vehicle (DMSO), or with erlotinib (E1000) or PHA665752 (PHA1000) at 1 µM for 24 h to inhibit EGFR or MET, respectively. Immunoblots were probed with different antibodies or p85a SH2 domain as indicated on right. Tyrosine phosphorylation and binding of p85A SH2 domain to proteins in the MW194 bin was strongly inhibited by erlotinib in erlotinib-sensitive cells (red arrows), while tyrosine phosphorylation and binding of p85A SH2 domain to proteins in the MW148 bin was strongly inhibited by PHA665752 (blue arrows). (C) HCC827 cell lysates were immunoprecipitated twice with antibody to MET (top) or EGFR (bottom). Lysate before immunoprecipitation (PreIP), first immunoprecipitate (P1), second immunoprecipitate (P2), and cleared lysate after immunoprecipitation (postIP) were immunoblotted with anti-MET or anti-EGFR antibodies or probed with p85a SH2 domain as indicated.
(9.02 MB TIF)

Figure S4 Changes in SH2 profiles following TKI treatment. (A) Median fold change (FC) and the median absolute deviation (MAD) for all SH2 domains whose binding significantly changed (p< = 0.125) by rosette assay after treatment with EGFR TKI. (B) Heat maps of far-Western binding data for each cell line treated with each tyrosine kinase inhibitor. Green indicates reduction in SH2 domain binding while red indicates increase in SH2 domain binding after TKI treatment. Each SH2 domain used for profiling is listed in columns and molecular weight bin is listed in rows.
(9.80 MB TIF)

Figure S5 Correlation of changes in SH2 profiles following TKI treatment. (A) Heat map of log2 fold changes (TKI-treated vs. untreated) from hierarchical clustering. Results for rosette are shown on left and far-Western blotting on right. (B) Pearson's correlation matrix shows overall similarity in fold changes between different cell lines and different treatments (compared to untreated cells; D, dasatinib-treated; E, erlotinib treated) for both the rosette and far-Western assays.
(7.04 MB TIF)

Figure S6 Preprocessing of the rosette data (referred to in Supplemental Methods). (A) Examining potential batch effects in the rosette assay. (B) Coefficient of Variation (CV) expressed as mean of the CV calculated for each cell line. (C) Histogram of signal level for the Abl domain. The histogram suggests intensities are not normally distributed. (D) Histogram of p values from the Shapiro test for normality. Most probes do not appear to be normally distributed. (E) Histograms of normally distributed SH2 domains. Histogram of Vav3 (left) and Rin1 (right) signal, with negative (green) and positive (red) controls indicated. (F) Histogram of differences in positive and negative controls across probes. 13 domains have differences <0 (white bar). (G) Histogram of domains with small differences between the positive and negative controls. Many are near 0. (H) Multidimensional Scaling (MDS) scatter plots of samples using (left) all domains, (right) filtered domains. Positive control (red) and negative control (green) are indicated. Similarity of samples is represented as pairwise distances. There does not appear to be any significant change in the relationship between samples as a result of filtering "noisy" probes.
(6.62 MB TIF)

Figure S7 Processing of far-Western blotting data (referred to in Supplemental Methods). (A) Alignment and quantification of multiple far-Western blotting results. Blot images from same gel were aligned using a frame marker of reference shots taken under normal white light during image scanning (Frame alignment). The aligned images were linked (Aligned lock) and further aligned with images from different gels using corresponding anti-phosphotyrosine Western blots. (B) Far-Western images were captured and partitioned into 20 grid elements (bins) per lane. Rows were numbered from the largest molecular weight (256–291 kDa) to smallest (<22 kDa). (C) Mapping of far-Western grid row and molecular weight. (D) Correlation between far-Western molecular weight bins across cell lines, between replicates. (E) Boxplot of far-Western signal for bins with correlation coefficients less than 0.5 between replicates. In most cases poor correlation is due to low signal.
(9.67 MB TIF)

Movie S1 SH2 far-Western blot results. Images of far-Western blotting results are compiled as a movie. SH2 domain binding experiments were performed on lysates of untreated (first half of the movie) and TKI-treated (second half of the movie) lung cancer cell lines. After correction for blot-to-blot variation (as shown in Figure S3), replica blots from two separate experiments were used for quantification of 20 bins per gel lane. Red grid lines indicate the location of bins used for quantification; pink line on top indicates EGFR-mutated cells. Approximate molecular size (in kDa) indicated in blue for each bin.
(14.14 MB MOV)
