# Supplementary material for: Characterizing Tyrosine Phosphorylation Signaling in Lung Cancer Using SH2 Profiling
Source: PLoS One. 2010 Oct 19;5(10):e13470. doi: 10.1371/journal.pone.0013470 (PMC2957407; doi:10.1371/journal.pone.0013470)
Supplement: Supplemental Methods S1 — Supplemental methods and references. (0.05 MB DOC) [file pone.0013470.s001.doc]

**SUPPLEMENTAL METHODS S1**

**Preprocessing of the rosette data and statistical analysis**

Each probe was quantified four times in two independent experiments. Therefore, the quantified intensity for each probe was analyzed for batch effects using ANOVA techniques and the coefficient of variation (CV) was calculated for each probe across the 4 replicates. Individual probes (n=96) were characterized using a positive and negative control and probes with low signal and small differences between controls (n=26) were excluded from clustering analysis (see below for details). Negative control was phosphatase-treated lysate, positive control was mixed lysates from pervanadate-treated cell lines; see Suppl. Ref. [2] for details of these controls. Hierarchical clustering was performed on the 70 probes (after median centering) using full linkage and uncentered correlation using Cluster 3.0 and Java Treeview.

*Batch effects.* The experiments consisted of two independent experiments, each performed in duplicate. We used the positive control sample as the example for exploring any batch effects and began by considering the first probe, Abl. As can be seen in Suppl. Fig. S6A, there appears to be a batch effect for this probe in the positive control sample between Experiment #1 and #2. Examining this further, we performed an ANOVA in the R statistical software package attempting to explain variance in observations using the experimental condition (treated or untreated), replicate number and sample group as variables. For the positive control sample, the experimental condition was an explanatory variable (p<0.05) for 48 of the 96 SH2 domain probes. In contrast, only 3 probes were significant (p<0.05) for different replicates and 14 were significant for the sample group. Surprisingly, testing for the same effect in the HCC827 cell line (across all SH2 domains), there were only 9 SH2 probes in which the experimental condition was a significant factor. By comparison there were 5 probes in which the replicate was a significant factor. As a result of this analysis, we decided to avoid normalizing or correcting the SH2 domain signal data for experimental batch effects. The mean of the 4 experiments was used for further analysis.

*Coefficient of variation.* The coefficient of variation was calculated for each SH2 probe, across samples. This was done by first comparing the CV from the four replicate values (two experiments, each with two replicates). The CV was combined across cell lines by taking the mean value of CV's for the probe. The mean CV values (shown in Suppl. Fig. S6B) indicate that most probes have a reasonable level of variability (across cell lines) using this assay.

*Rosette SH2 domain probe analysis.* We examined the properties of the SH2 domains across cell lines to further characterize the signal from these probes. Suppl. Fig. S6C is a histogram of signal for the Abl SH2 domain. This suggests that the signal data are not normally distributed, but rather high signal is a rare event. Therefore we applied the Shapiro test for normality to all of the SH2 probes. Suppl. Fig. S6D illustrates a histogram of p values from the Shapiro test indicating that most of the SH2 domains are not normally distributed, as defined by the test. Four SH2 domains (Nsp3, Rin 1, Syk (NC) and Vav3) are unusual in that the signal from these probes appears to be normally distributed. Histograms for two of these domains are shown in Suppl. Fig. S6E, with the negative and positive controls graphed in green and red, respectively. Note the overall signal levels are somewhat low, suggesting there is little specific signal and thus noise may drive the values derived. A box plot of these SH2 domains further suggests that the four domains identified have low signal levels for all cell lines measured (not shown).

*Positive and negative controls.* Next the positive and negative control samples were analyzed to better understand the SH2 domain signals. Suppl. Fig. S6F is a histogram of differences between positive and negative controls across domains. There is a wide range of differences, although 13 domains have differences less than 0 (i.e. the negative control has higher signal than the positive control). This is likely due to low specific signal leading to relatively high contribution of noise. Most SH2 domains had relatively few signals outside the expected range of positive/negative control signals. Therefore, we believe the controls are accurate indicators of signal range in most instances.

Next we further examined the SH2 domains with small (or negative) differences between the positive and negative controls. The minimum difference in positive and negative controls is Dapp1, with a -2.08125 difference. Examining the histogram (shown in Suppl. Fig. S6G) suggests that domains with no signal are centered about zero (no difference in positive and negative controls) and extend to a difference of approximately 2.1. This suggests that 26 of the domains may not be informative. The mean maximum signal value across these 26 domains is 2.03, consistent with little or no specific signal.

MDS plots (shown in Suppl. Fig. S6H) of cell lines across all SH2 domains (left) or only the “good” domains (filtered by differences in positive-negative controls < 2.1, right) indicate that the overall patterns in the data are not significantly changed by removing these probes (as expected since the signal values are very low). As a result, these probes were not included in the global (clustering): Abl (R174K); SHE; CblC; SHF; Chimerin1; SHP-1(NC); Cis1; Slap; Dapp1; Slp76; Nsp3; Socs1; Rin1; Stat1; Rin2; Stat5A; Rin3; Supt6h; SH2A; Syk(NC); ShcC; Vav3; ShcD; Zap70(NC); Shd; and GST.

**Processing far-Western blotting data**

*Alignment and quantification of multiple far-Western blotting results.* Two independent far-Western analyses were performed on the lung cancer cell lines. The following method was used to quantify comparable bands on multiple blots from different experiments. In far-Western experiments, replicas of blots were probed with a first set of SH2 domains and stripped membranes were repeatedly used for reprobing with additional SH2 probes. Blot images derived from the same membrane share identical shape, while there is a minor variability comparing different membranes. To align blot images with identical shape, “reference shots” were taken under white light for every chemiluminescence scan. Reference shots and SH2 blot images were then set as multiple layers in Adobe Photoshop CS3 software, and aligned by scaling and rotating. Aligned images were linked for further image alignment (Suppl. Fig. S7A, Frame alignment and Aligned lock). For blot images derived from different gels, anti-phosphotyrosine Western blotting was performed as a reference and their images were also imported into Photoshop. Each linked group of images was aligned with other groups by slightly distorting using the reference phosphotyrosine blots as a guide (Band alignment). Finally, the aligned images were exported to ImageJ (v1.40) for densitometry as an image sequence and partitioned into 20 grid elements per lane as shown in Suppl. Fig. S7B,C. The image sequence was background-subtracted and batch quantified using a custom-made plug-in (Slot Reader). A full description of the methodology will be published elsewhere.

*Correlation between far-Western replicates.* Since there were two replicates used in the far-Western experiment, we examined the correlation between replicates for each row, or molecular weight bin, across cell lines. The Pearson correlation was used for each of the 20 bins, and for each of the 36 SH2 domains assayed. Suppl. Fig. S7D shows a histogram of the correlation coefficients across the 720 unique bins between far-Western replicates. As can be seen from the figure, 608 of the 720 bins have correlation coefficients > 0.5. Further examining the domains with negative (or small) correlation coefficients, Suppl. Fig. S7E illustrates that for most of these bins the signal is very low, suggesting background noise plays a large part in variability for these probes. As a result, the data was combined across replicates using the mean. For analysis, data was preprocessed in the following way. After examination of the far-Western images, for many bins signal was very faint and not considered to be informative. Suppl. Fig. S7E shows a boxplot of far-Western signal for bins with correlation coefficients less than 0.5 between replicates. The figure indicates that most of the poor correlation occurs due to low signal. As a result, we calculated the 25th percentile of intensities across the experiment (3.753551) and used this value as the minimum signal.

For comparison of TKI response (Fig. 7C, Suppl Fig. S5A), hierarchical clustering was performed on log_2_ fold change values without normalization and using full linkage and uncentered correlation. Rosette probes and Far-Western bands were not filtered for clustering.

**Statistical analysis**

*Two-group comparisons.* Domains were identified as statistically significant with respect to dichotomous characteristics (EGFR Mutation, RAS Mutation or Met Activation) using a Mann-Whitney test applied to each domain. To correct for multiple testing problems, false discovery rates (q values) were calculated using the Q Value package in Bioconductor [3] and a 10% FDR (q ≤ 0.1) was considered significant. Bar graphs showing differences between groups were displayed using mean and standard errors (e.g., Figs. 3A and 6A).

*Permutation tests.* To identify probability of a clustering pattern occurring by chance, 100,000 permutations of cell lines were randomly arranged and the number of times a cluster consists of k or more cells with particular characteristics was calculated.

*Correlation to erlotinib sensitivity*. Erlotinib IC_50_ values for the 22 cell lines were ln transformed and the Pearson's correlation coefficient was computed for each domain. Domains were considered correlated with |R|≥0.5 (R^2^ ≥ 0.25).

*Far-Western clustering-untreated cells.* 720 domain-specific bands (20 bands x 35 SH2 domains) were filtered to retain those with a standard deviation > 5, and at least 11 of 22 cell lines having intensity above 5.0 for a specific domain-specific band. Hierarchical clustering was performed on the remaining 188 bands using full linkage and uncentered correlation using Cluster 3.0 and visualized using Java Treeview. No normalization was used in clustering, but for visualization purposes, the intensities were median-centered so that green represents values below the median probe value and red above.

*Far-Western differences with tyrosine kinase inhibitors.* Data from individual bins was quantified. Binding data for 31 probes (620 bands) were available for the TKI inhibitor studies and used to calculate fold changes between treated and untreated cells. This data was not normalized or filtered (Suppl. Fig. S4B).

*Characterizing changes with treatment.* The same analytical process was used to calculate SH2 signal as above. Differences in signal were quantified as the log_2_ fold change (log_2_(treated/untreated)). A paired statistical test was performed for each domain; however since the data does not always follow a normal distribution (tested using the Shapiro-Wilk test for normality) the Wilcoxon signed rank test was used instead. This test, together with the small sample size, leads to only 5 distinct p values possible. Therefore, in addition to a p value filter (p=0.125 is the minimum possible p value) we also considered fold change. All domains with p=0.125 and a median fold change (across the four cell lines) of at least 1.5 were considered as significant (shown in Suppl. Fig. S4A).

**SUPPLEMENTAL REFERENCES**

1. Dierck K, Machida K, Mayer BJ, Nollau P (2009) Profiling the tyrosine phosphorylation state using SH2 domains. Meth Mol Biol 527: 131-155.

2. Machida K, Thompson CM, Dierck K, Jablonowski K, Kärkkäinen S, et al. (2007) High-throughput phosphotyrosine profiling using SH2 domains. Mol Cell 26: 899-915.

3. Storey JD, Tibshirani R (2003) Statistical significance for genome-wide studies. Proc Natl Acad Sci USA 100: 9440-9445.
