# Supplementary material for: Characterizing Tyrosine Phosphorylation Signaling in Lung Cancer Using SH2 Profiling
Source: PLoS One. 2010 Oct 19;5(10):e13470. doi: 10.1371/journal.pone.0013470 (PMC2957407; doi:10.1371/journal.pone.0013470)
Supplement: Table S1 — Characteristics of cell lines used in this study. Adeno = adenocarcinoma; Squamous = squamous cell carcinoma; BAC = bronchiolioalveolar carcinoma; Large = large cell carcinoma; NOS = not otherwise specified; WT = wildtype. (0.06 MB DOC) [file pone.0013470.s002.doc]

| Cell | Histology | EGFR Status | KRAS Status | Other | IC_50_(nM) Erlotinib |
| --- | --- | --- | --- | --- | --- |
| HCC827 | Adeno | del exon 19 | WT |  | 3.69 |
| PC9 | Adeno | del exon 19 | WT |  | 7.5 |
| H4006 | Adeno | del exon 19 | WT |  | 2.79 |
| H1650 | Adeno | del exon 19 | WT | PTEN loss | 12700 |
| H1975 | Adeno | L858R/T790M | WT | T790M | 5600 |
| H2279 | Adeno | del exon 19 | WT |  | 5300 |
| H820 | Adeno | del exon 19 | WT | T790M, MET | >2000 |
| H292 | Squamous | WT | WT |  | 63.4 |
| H358 | BAC | WT | Mut exon1 |  | 199.1 |
| H441 | Adeno | WT | Mut exon2 |  | 6290 |
| A549 | Adeno | WT | Mut exon1 |  | 10970 |
| H460 | Large Cell | WT | Mut exon2 |  | 8880 |
| H1299 | Large Cell | WT | WT |  | 8300 |
| H1648 | Adeno | WT | WT |  | 75.8 |
| H2122 | Adeno | WT | Mut exon1 |  | 2840 |
| H226 | Squamous | WT | WT |  | 45400 |
| H157 | Squamous | WT | Mut exon1 |  | 19600 |
| H322 | BAC | WT | WT |  | 135.1 |
| H23 | Adeno | WT | Mut exon1 |  | 7800 |
| H596 | Adeno/Squam | WT | WT |  | 5000 |
| H2172 | NOS | WT | WT |  | 11900 |
| UK29 | Adeno | WT | Mut exon2 |  | 2200 |

| Class | IC_50_(nM) Erlotinib |
| --- | --- |
| positive (sensitive) | <10 |
| Intermediate (moderate) | 10-1000 |
| negative (insensitive) | >1000 |

**Supplementary Table S1**
