# Supplementary figures and images for: Characterizing Tyrosine Phosphorylation Signaling in Lung Cancer Using SH2 Profiling
Source: PLoS One. 2010 Oct 19;5(10):e13470. doi: 10.1371/journal.pone.0013470 (PMC2957407; doi:10.1371/journal.pone.0013470)

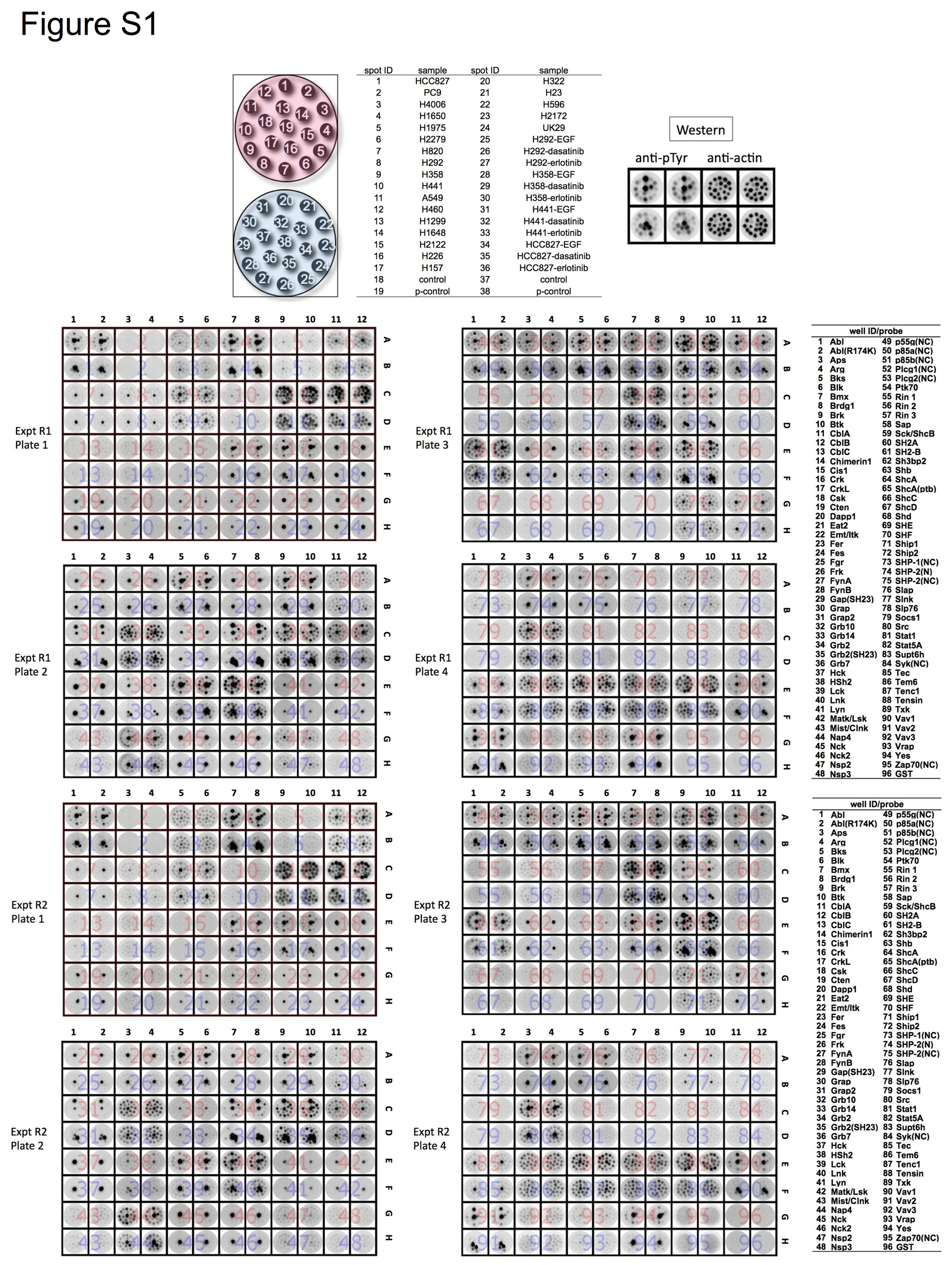

Supplement: Figure S1 — SH2 rosette assays. The SH2 rosette assay (high throughput dot blotting) was performed as described [1], [2]. Lung cancer and control cell lysates were spotted in duplicate on nitrocellulose membrane as indicated (top) in register with wells of a 96-well chamber plate. Each well was separately incubated with GST-SH2/PTB domain, GST control, anti-phosphotyrosine antibody, or anti-actin antibody (probe list is on the right). Control = negative control (phosphatase-treated lysate); p-control = positive control (mixed lysates from pervanadate-treated cell lines); see [2] for details. Two independent experiments were performed in duplicate, providing four quantifiable data points for each probe/sample pair. Array images were background-subtracted and the integrated density of each spot was measured using ImageJ densitometry (v1.40). (5.97 MB TIF) [file pone.0013470.s005.tif]

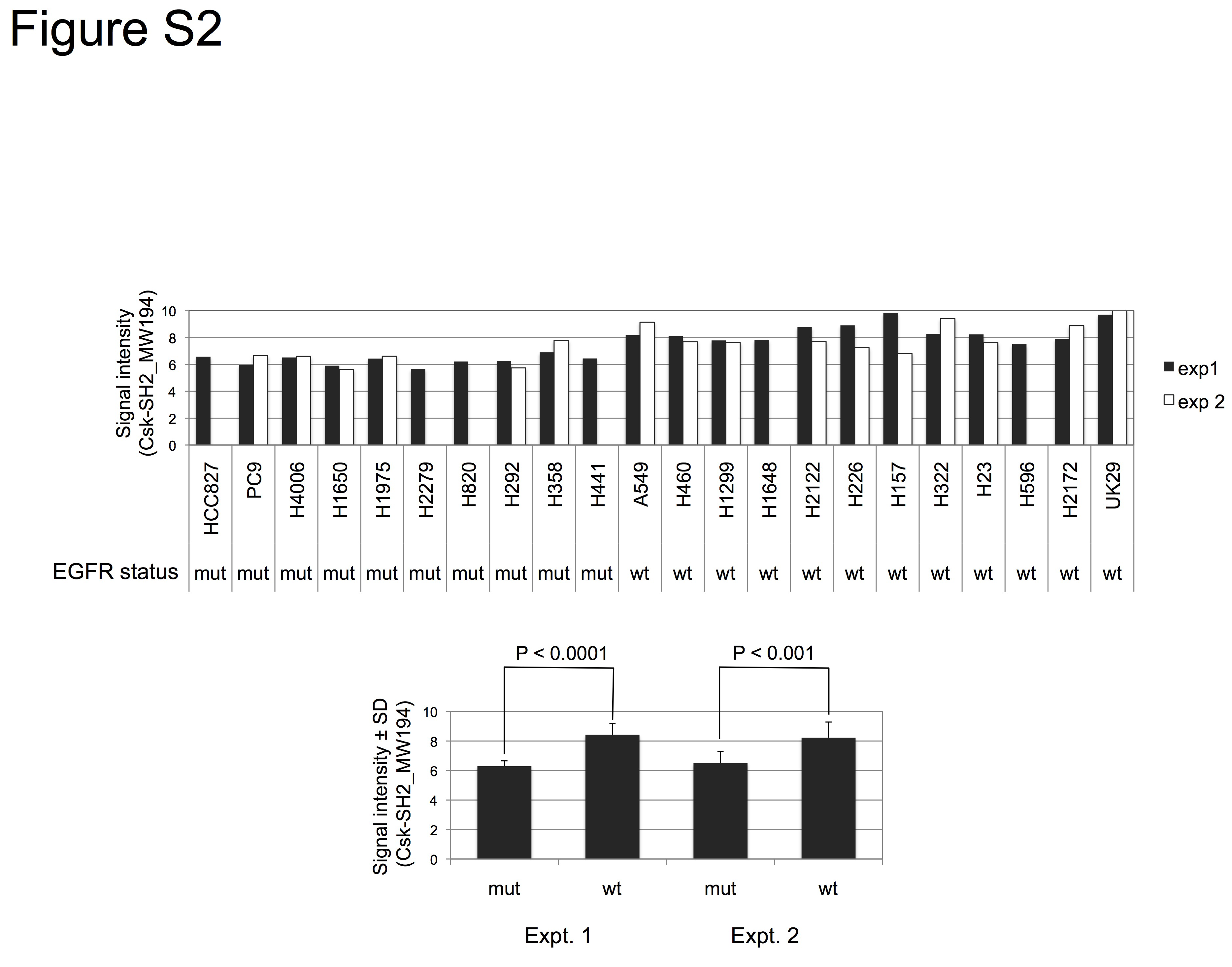

Supplement: Figure S2 — Correlation of Csk SH2 binding and EGFR mutation status. Top: Signal intensity from far-Western analysis using CSK SH2 probe was quantified in multiple blots. Black bars represent average of two independent blots (Expt. 1; these data are provided in Suppl. Table S2 and were used for clustering analysis). White bars represent data from an independent blot performed at another time with a subset of cell lines. EGFR mutation status of cell lines (mut = mutant, wt = wild-type) is indicated below. Bottom: Average CSK signal intensity for mutant vs. wt cell lines. Differences are statistically significant by unpaired T test as indicated by P values. (1.64 MB TIF) [file pone.0013470.s006.tif]

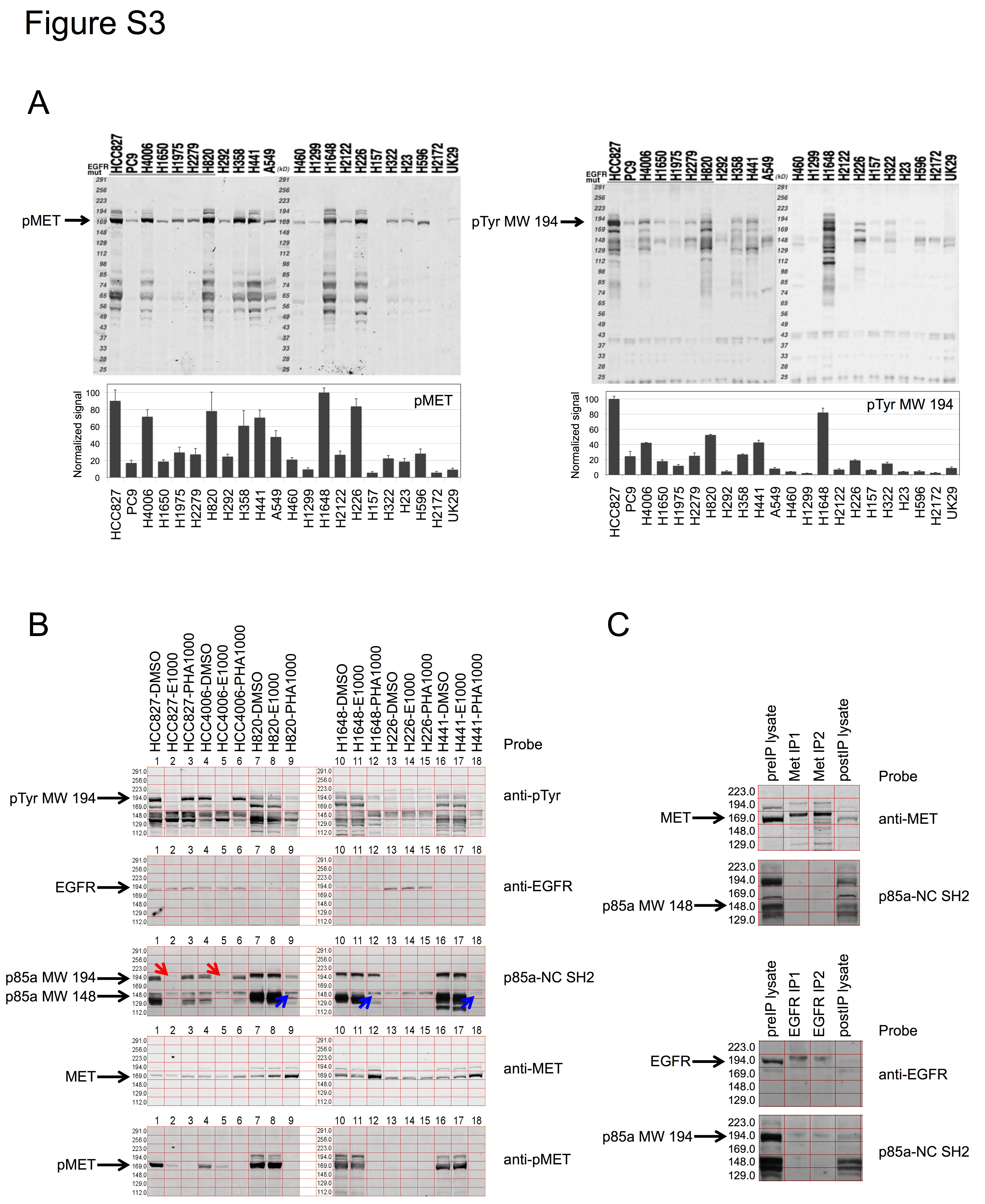

Supplement: Figure S3 — MET and EGFR family activation. (A) Left: Lysates from lung cancer cell lines were separated by SDS-PAGE, transferred to membranes, and blotted with phosphospecific anti-MET antibody (recognizing pY1334/1335). Signal was detected by chemiluminescence and the pMET band (arrow) was quantified using ImageJ densitometry. Representative blot (upper) and quantified result from multiple experiments (lower) are shown. Right: Similarly, protein bands of approximately 194 kDa on anti-pTyr blots (at molecular weight of EGFR family members, arrow) were quantified. Values are given as percent of maximum signal. (B) Cell lines indicated were treated with vehicle (DMSO), or with erlotinib (E1000) or PHA665752 (PHA1000) at 1 µM for 24 h to inhibit EGFR or MET, respectively. Immunoblots were probed with different antibodies or p85a SH2 domain as indicated on right. Tyrosine phosphorylation and binding of p85A SH2 domain to proteins in the MW194 bin was strongly inhibited by erlotinib in erlotinib-sensitive cells (red arrows), while tyrosine phosphorylation and binding of p85A SH2 domain to proteins in the MW148 bin was strongly inhibited by PHA665752 (blue arrows). (C) HCC827 cell lysates were immunoprecipitated twice with antibody to MET (top) or EGFR (bottom). Lysate before immunoprecipitation (PreIP), first immunoprecipitate (P1), second immunoprecipitate (P2), and cleared lysate after immunoprecipitation (postIP) were immunoblotted with anti-MET or anti-EGFR antibodies or probed with p85a SH2 domain as indicated. (9.02 MB TIF) [file pone.0013470.s007.tif]

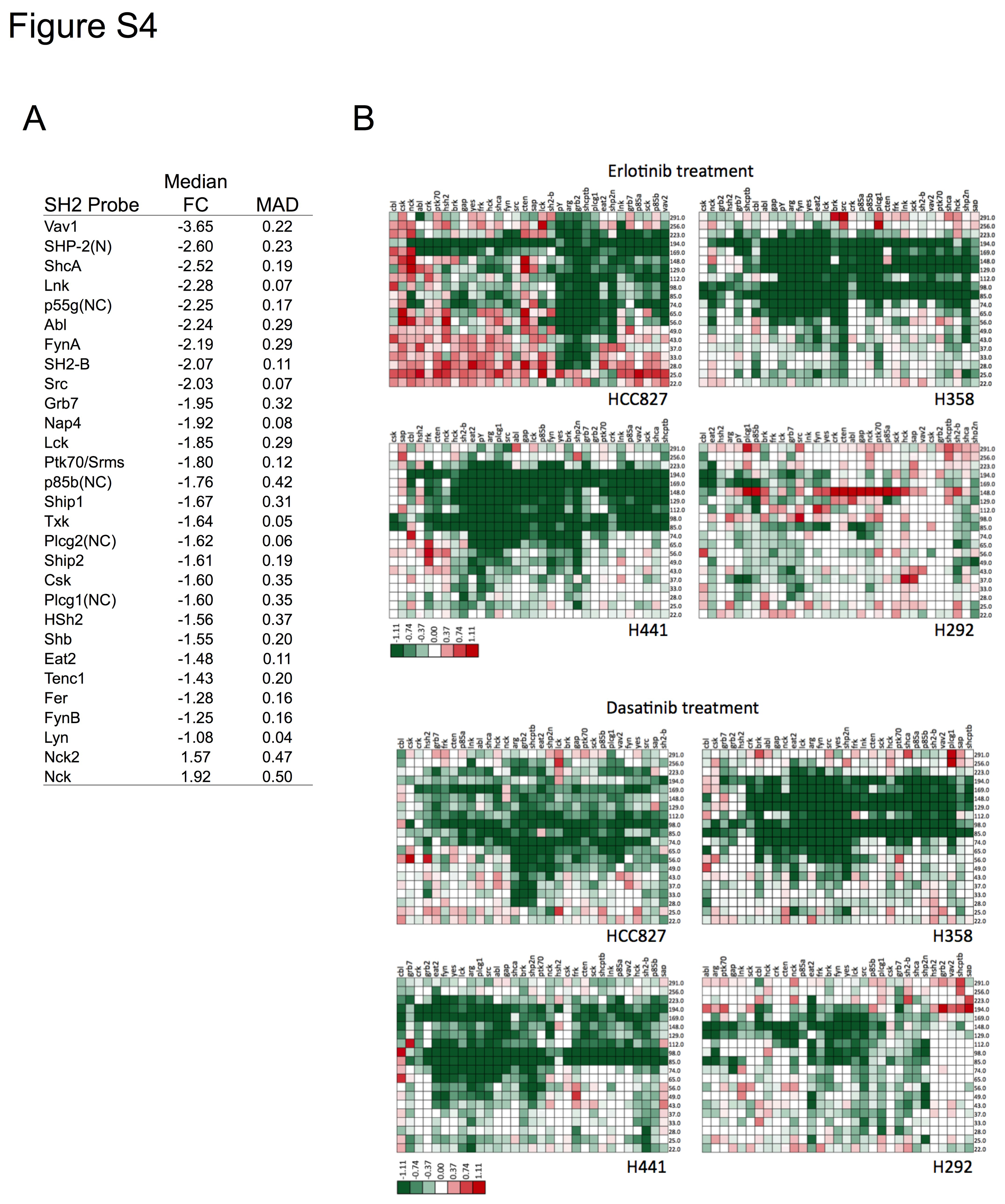

Supplement: Figure S4 — Changes in SH2 profiles following TKI treatment. (A) Median fold change (FC) and the median absolute deviation (MAD) for all SH2 domains whose binding significantly changed (p< = 0.125) by rosette assay after treatment with EGFR TKI. (B) Heat maps of far-Western binding data for each cell line treated with each tyrosine kinase inhibitor. Green indicates reduction in SH2 domain binding while red indicates increase in SH2 domain binding after TKI treatment. Each SH2 domain used for profiling is listed in columns and molecular weight bin is listed in rows. (9.80 MB TIF) [file pone.0013470.s008.tif]

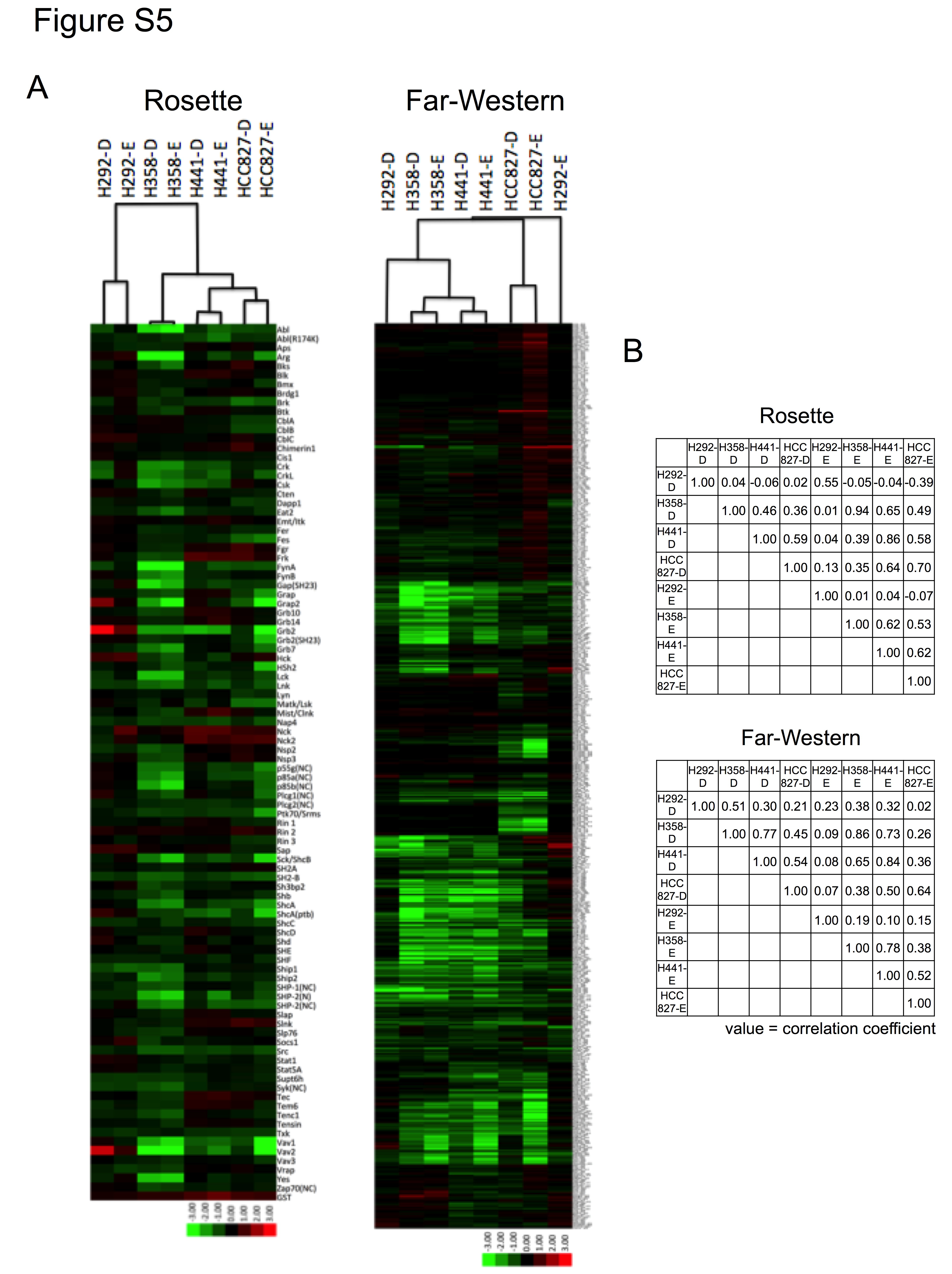

Supplement: Figure S5 — Correlation of changes in SH2 profiles following TKI treatment. (A) Heat map of log2 fold changes (TKI-treated vs. untreated) from hierarchical clustering. Results for rosette are shown on left and far-Western blotting on right. (B) Pearson's correlation matrix shows overall similarity in fold changes between different cell lines and different treatments (compared to untreated cells; D, dasatinib-treated; E, erlotinib treated) for both the rosette and far-Western assays. (7.04 MB TIF) [file pone.0013470.s009.tif]

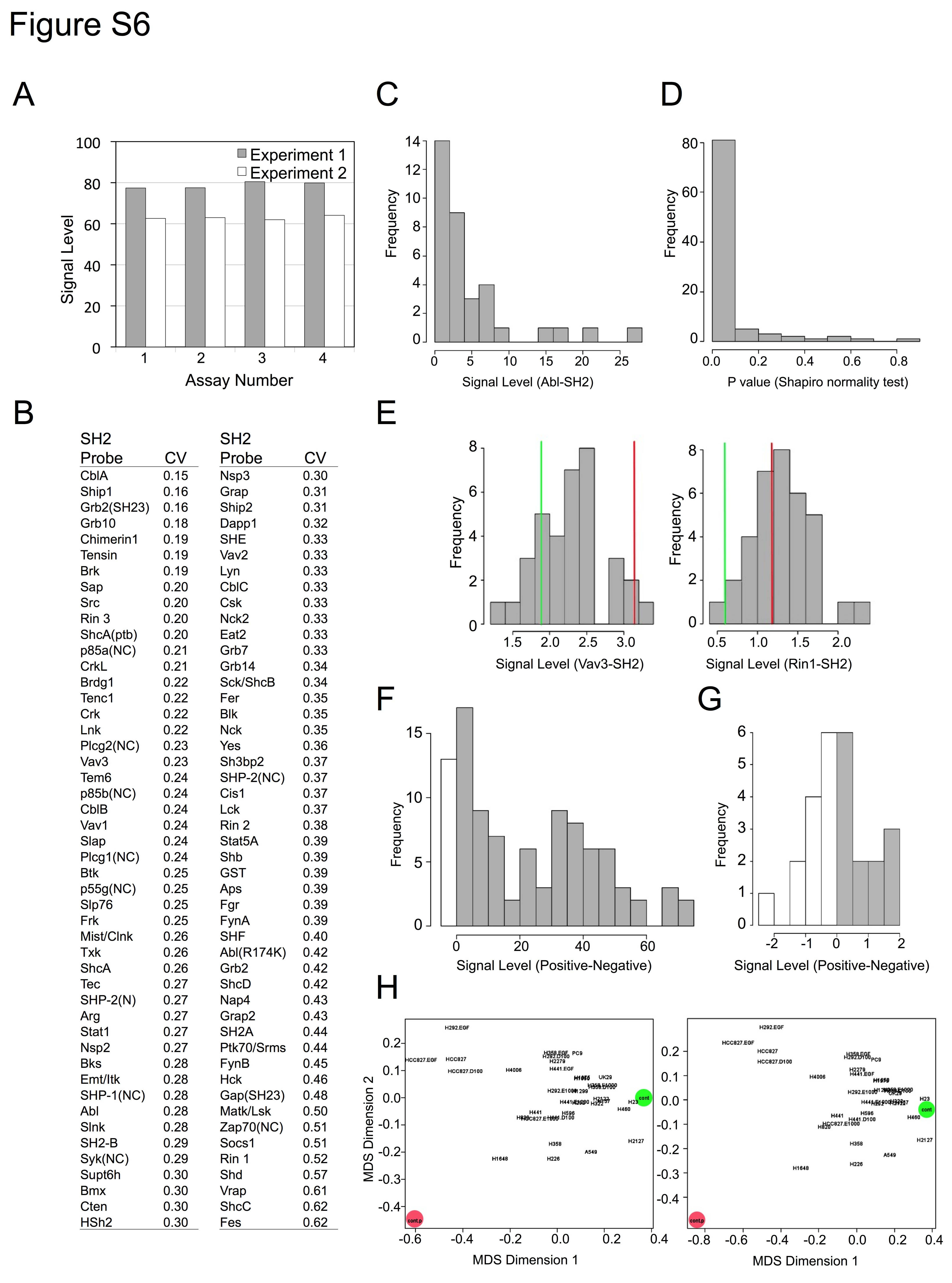

Supplement: Figure S6 — Preprocessing of the rosette data (referred to in Supplemental Methods). (A) Examining potential batch effects in the rosette assay. (B) Coefficient of Variation (CV) expressed as mean of the CV calculated for each cell line. (C) Histogram of signal level for the Abl domain. The histogram suggests intensities are not normally distributed. (D) Histogram of p values from the Shapiro test for normality. Most probes do not appear to be normally distributed. (E) Histograms of normally distributed SH2 domains. Histogram of Vav3 (left) and Rin1 (right) signal, with negative (green) and positive (red) controls indicated. (F) Histogram of differences in positive and negative controls across probes. 13 domains have differences <0 (white bar). (G) Histogram of domains with small differences between the positive and negative controls. Many are near 0. (H) Multidimensional Scaling (MDS) scatter plots of samples using (left) all domains, (right) filtered domains. Positive control (red) and negative control (green) are indicated. Similarity of samples is represented as pairwise distances. There does not appear to be any significant change in the relationship between samples as a result of filtering "noisy" probes. (6.62 MB TIF) [file pone.0013470.s010.tif]

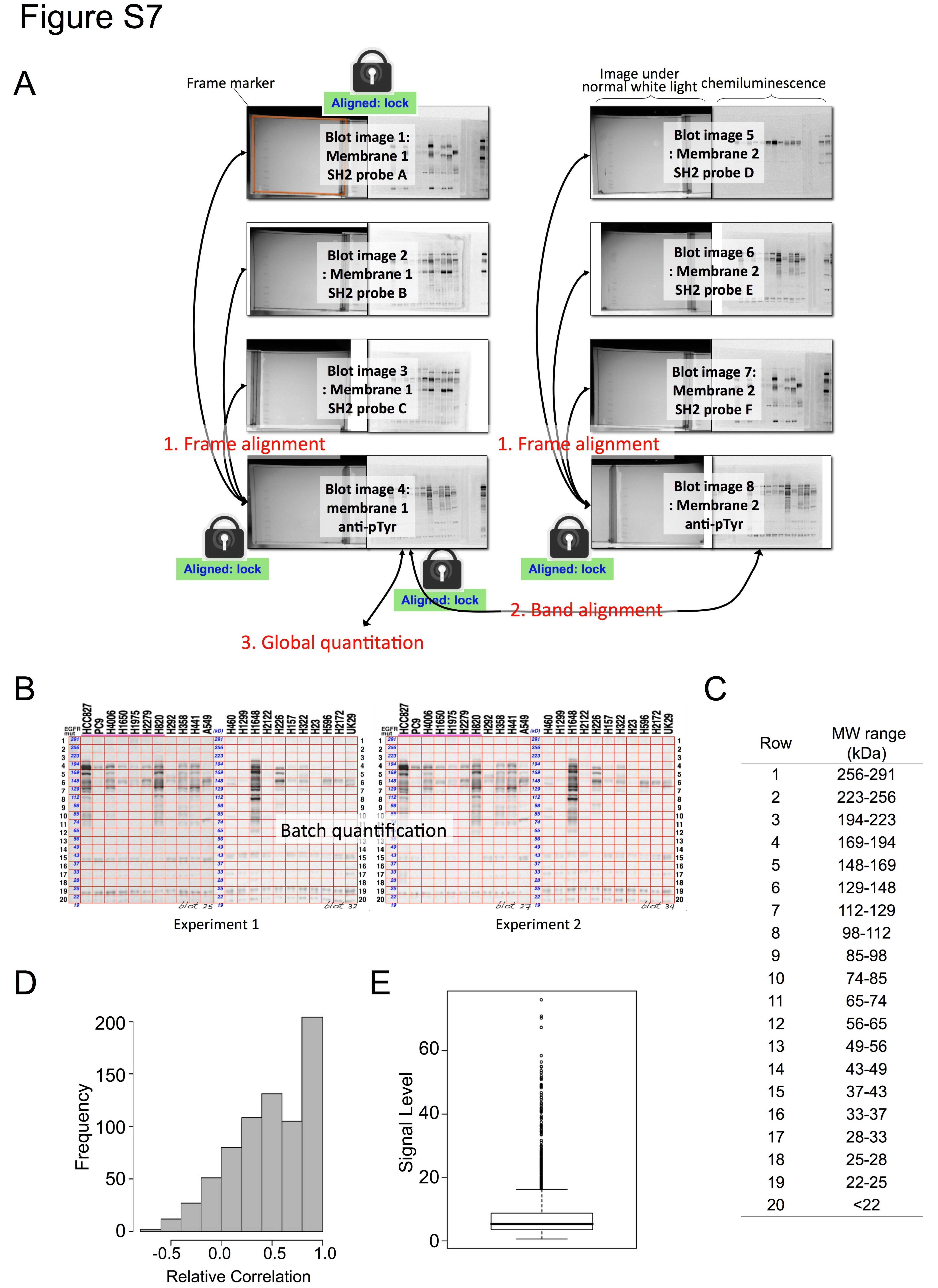

Supplement: Figure S7 — Processing of far-Western blotting data (referred to in Supplemental Methods). (A) Alignment and quantification of multiple far-Western blotting results. Blot images from same gel were aligned using a frame marker of reference shots taken under normal white light during image scanning (Frame alignment). The aligned images were linked (Aligned lock) and further aligned with images from different gels using corresponding anti-phosphotyrosine Western blots. (B) Far-Western images were captured and partitioned into 20 grid elements (bins) per lane. Rows were numbered from the largest molecular weight (256–291 kDa) to smallest (<22 kDa). (C) Mapping of far-Western grid row and molecular weight. (D) Correlation between far-Western molecular weight bins across cell lines, between replicates. (E) Boxplot of far-Western signal for bins with correlation coefficients less than 0.5 between replicates. In most cases poor correlation is due to low signal. (9.67 MB TIF) [file pone.0013470.s011.tif]
